# An Overview of Stakeholders, Methods, Topics, and Challenges in Participatory Approaches Used in the Development of Medical Devices: A Scoping Review

**DOI:** 10.34172/ijhpm.2022.6839

**Published:** 2022-11-05

**Authors:** Kas Woudstra, Rob Reuzel, Maroeska Rovers, Marcia Tummers

**Affiliations:** ^1^Department of Health Evidence and Operation Rooms, Radboud University Medical Center, Nijmegen, The Netherlands.; ^2^Department of Health Evidence, Radboud University Medical Center, Nijmegen, The Netherlands.

**Keywords:** Stakeholder Engagement, Participatory Research, Medical Device Development, User-Centred Design, Public Participation

## Abstract

**Background:** There is a wide variety of participatory approaches to involve stakeholders in the development of medical devices, but there is no comprehensive overview of these approaches. We therefore studied what participatory approaches are used in the development of medical devices as well as the most important characteristics and challenges of these approaches.

**Methods:** We conducted a scoping review and searched PubMed, Embase and Web of Science for articles published between July 2014 and July 2019. Papers were included if they presented original research featuring any form of stakeholder participation in the development of medical devices. We used The Spectrum of Public Participation to categorise the approach of each paper. Subsequently, we described the characteristics of each approach: the stakeholders involved, data collection methods, and topics addressed. We also identified challenges of the approaches as reported by researchers.

**Results:** 277 papers were included, which could be categorised into three levels of participation: collaboration, involvement, and consultation. Patients and healthcare professionals are frequently engaged in all approaches. The most often used methods are workshops in the collaboration approach papers, and interviews in the involvement and consultation approach papers. Topics addressed in all approaches are: the problem, device requirements, design choices, testing, and procedural aspects of involvement. Reported challenges entail issues related to sampling, analysis, social dynamics, feasibility, and the limited number of topics that can be addressed.

**Conclusion:** Participatory approaches reported in literature can be categorised in three overarching approaches that have comparable methodological characteristics. This suggests that if researchers want to apply a participatory approach it is not necessary to adopt a pre-determined approach, such as ‘participatory action research’ (PAR). Instead, they can independently determine the degree of participation, stakeholders, methods, topics, and strategies to account for challenges, making sure the participatory approach fits their research question and context.

## Background

 Stakeholder engagement in medical device development is increasing. It is promoted by various governmental institutions like the European commission,^1^ National Health Service,^2^ and Food and Drug Administration.^3^ The European Medical Device Regulation took effect in 2020 and recommends to perform clinical evaluations by clinical stakeholders in the early phases of medical device development, to ensure safety and functionality.^4^ In addition, various medical device companies endorse stakeholder engagement.^5,6^ The idea is that by involving stakeholders devices are aligned with needs of people with vested interests, usability and functionality of devices are improved, and the overall productivity of the development process is increased.^7-9^

 Although stakeholder engagement increased in medical device development, it finds its origin in different participatory research traditions. Three dominant approaches can be distinguished: user-centred design, participatory design, and participatory action research (PAR). User-centred design is a consolidated research approach which emerged in the 1970s. It is widely used in technology development to improve interactions between people and devices by focussing on users’ experiences and needs.^10,11^ Participatory design arose in the 1960s in Scandinavia, and is based on cooperation between designers and users.^12,13^ PAR emerged to empower minorities in the civil and indigenous rights movements, ecological activism, education, and medicine. There are different types of action research which all have in common that non-experts participate in all aspects of a research process, aiming to make improvements for these participants.^13-15^

 In recent years, different participatory approaches were applied in the setting of medical device development. Yet, the reviews that are available are limited to specific technologies, treatments, or participatory research approaches.^16-21^ Moore et al have for example published a systematic review of participatory methods, but this review only studied eHealth and the participation of health service users such as patients.^19^ An overview that is not limited to a specific participatory approach or a specific technology can be useful for researchers. It will provide insight in both the variety and similarities of reported participatory approaches for stakeholder involvement in medical device development. Furthermore, by analysing how participatory approaches are used *in practice*, it provides insight in how methods are used rather than how approaches differ theoretically. This can especially elucidate which methodological challenges occur in practice. The research question of our scoping review therefore is: which participatory approaches are used in the development of medical devices, and what are the most important characteristics and challenges of these approaches?

## Methods

 We performed a scoping review, which is similar to systematic reviews in that it follows a structured process and aims to offer a comprehensive overview of a body of literature, but is more suitable for the broad research aim of identifying and mapping methods like presented in this study.^22^ We used the Joanna Briggs Manual for Evidence Synthesis for Scoping reviews to inform the development of the search, selection, and data synthesis. The search, selection and synthesis therefore met standards for rigor in review literature and we have outlined these in our review protocol, see Supplementary file 1.^23,24^ Our scoping review comprised the following stages: (1) a methodical search of studies relevant to our research question, (2) a systematic study selection process using predetermined eligibility criteria, (3) the charting of relevant data reflected in included studies, using a data extraction tool, and (4) use of the completed data extraction tool to synthesize and interpret the results of our study.

###  Search Strategy

 With the assistance of professional librarians, we developed a strategy for searching PubMed, Web of Science and Embase for relevant literature published from July 1, 2014 to July 1, 2019. In PubMed and Embase, we used medical subject headings (MeSH) terms, Emtree subjects and keywords to capture the concepts of participatory approaches used in the development of medical devices. The studies had to report a stakeholder involvement approach for developing a medical device. Participatory approaches were defined as procedures that delegate decision-making power or influence to study participants.^9^ For medical devices, we used the definition of the World Health Organization (WHO), which defines a device as an ‘instrument, apparatus, implement, machine, appliance, implant, reagent for in vitro use, software, material or other similar or related article’ to be used in ‘diagnosing, preventing, monitoring, treating or alleviating’ disease and injury; or to control conception and sustain life.^25^ Studies were included that present original study data, were published in English, and published between July 1, 2014 and July 1, 2019. See Supplementary file 2 for our complete search strategy.

###  Study Selection

 Three researchers reviewed the first 100 titles and abstracts using Rayyan.^26^ Subsequently, two reviewers continued to review the next 500 titles and abstracts to increase the rigor of the study selection process. The titles and abstracts of the remaining papers were reviewed by one author, but in case of doubt a second reviewer was consulted. Full-text review of the selected studies were conducted again by two reviewers, and any disagreements were resolved in discussions with a third reviewer.

###  Data Extraction

 The lead author, together with two of the co-authors, developed a data extraction tool to capture relevant methodological characteristics from the included papers. We extracted and presented data on 13 data-items that are listed in Supplementary file 3. These range from the types of stakeholders, the data collection methods, the topics that are discussed in the involvements, and the challenges that the researchers experience themselves when applying the participatory approaches. The data extraction was performed by two reviewers. Any disagreements or doubts were solved in discussions between these authors.

###  Data Synthesis 

 We used the The Spectrum of Public Participation, a widely accepted frame for describing degrees of participation developed by the International Association for Public Participation, to categorise the participatory approach used in each paper.^27^ The spectrum distinguishes between five levels of participation: (1) ‘inform,’ where decision makers inform the public, (2) ‘consult,’ where public feedback on an analysis is obtained, (3) ‘involve,’ where the public in involved throughout a project to align decisions with public concerns, (4) ‘collaborate,’ where public members participate in decision-making, and (5) ‘empower,’ when decision-making is fully in public hands.^27^ This grouping is closely related to the well-known ‘citizen participation ladder’ by Arnstein, but uses more neutral and contemporary descriptions of the different degrees of participation.^28,29^

 Two authors (KW and MT) determined the grouping of the research approaches of the papers into the above-mentioned levels of participation, based on the description of the research approach as found in the articles. We calculated which percentage of papers per approach presented a specific type of stakeholders or data collection method: eg, the percentage of papers that involved patient, experts, etc. Topics and challenges as reported by the authors were extracted as free text to mark and describe meaningful passages in the transcripts. Subsequently, this text was coded and grouped into themes to create an overview of topics and challenges that are addressed in the included articles. This enabled us to calculate for each approach the percentage of papers in which specific topics and challenges were reported. These analyses were done by the first author and have been discussed with a second author. The PRISMA-ScR guideline was used for reporting our study.

## Results

 After deduplicating the records, the initial search identified 14 838 records, that provided 518 relevant articles after screening title and abstract. Following full text screening, 277 articles were included in the review. The PRISMA flow chart (Supplementary file 4) provides detailed information about the literature selection process.The characteristics and challenges of all individual articles are included in Supplementary file 5, and the reference list of all the included articles is included in Supplementary file 6. The majority of the studies are from Europe (n = 116, 42%) and North America (n = 102, 37%), followed by Oceania/Australia (n = 23, 8%), Asia (n = 16, 6%), South America (n = 5, 2%), and Africa (n = 7, 3%) or from multiple continents (n = 8, 3%). The majority of studies report electronic health (e-health) technologies (n = 243, 88%), a category under which we group all forms of health services that are delivered via websites, apps, or other internet and computing media. These e-health technologies are phone or tablet applications (n = 116, 48%), websites or computer programs (n = 57, 23%), e-health system combining apps, websites and computer programs (n = 53, 22%), virtual avatars (a computer animation of a person with which users can interact) (n = 5, 2%), games (n = 8, 3%), and a mobile device (n = 2, 1%). Other technologies are devices (n = 26, 9%) such as robotics, and tracking systems using sensors (n = 5, 3%) such as an equipment track and trace system.

###  Categorisation of the Participatory Approaches

 All participatory approaches could be classified into three levels of the spectrum of public participation: collaboration, involvement, and consultation. In collaboration, stakeholders are involved as equal partners in the decision-making process. Examples of collaboration approaches are co-design, co-creation, participatory design, community-based action research, and PAR. Seventy-five papers fall into this first category that we will name ‘collaboration approaches.’

 In involvement, stakeholders are involved to align decisions with their concerns. This level of participation is prevalent in all the approaches that use a ‘user-centred design’ or a variant of this approach. Ninety-eight papers are identified as such.

 In consultation, stakeholders are invited to inform specific decisions but are not involved in decision-making. Qualitative studies with eg, focus groups and interviews were categorized in this approach, together with studies that inform a subset of designing, such as usability studies. The consultation approach was identified in 104 papers.

###  Characteristics of the Three Participatory Approaches

####  Stakeholders Involved

**Figure 1:**
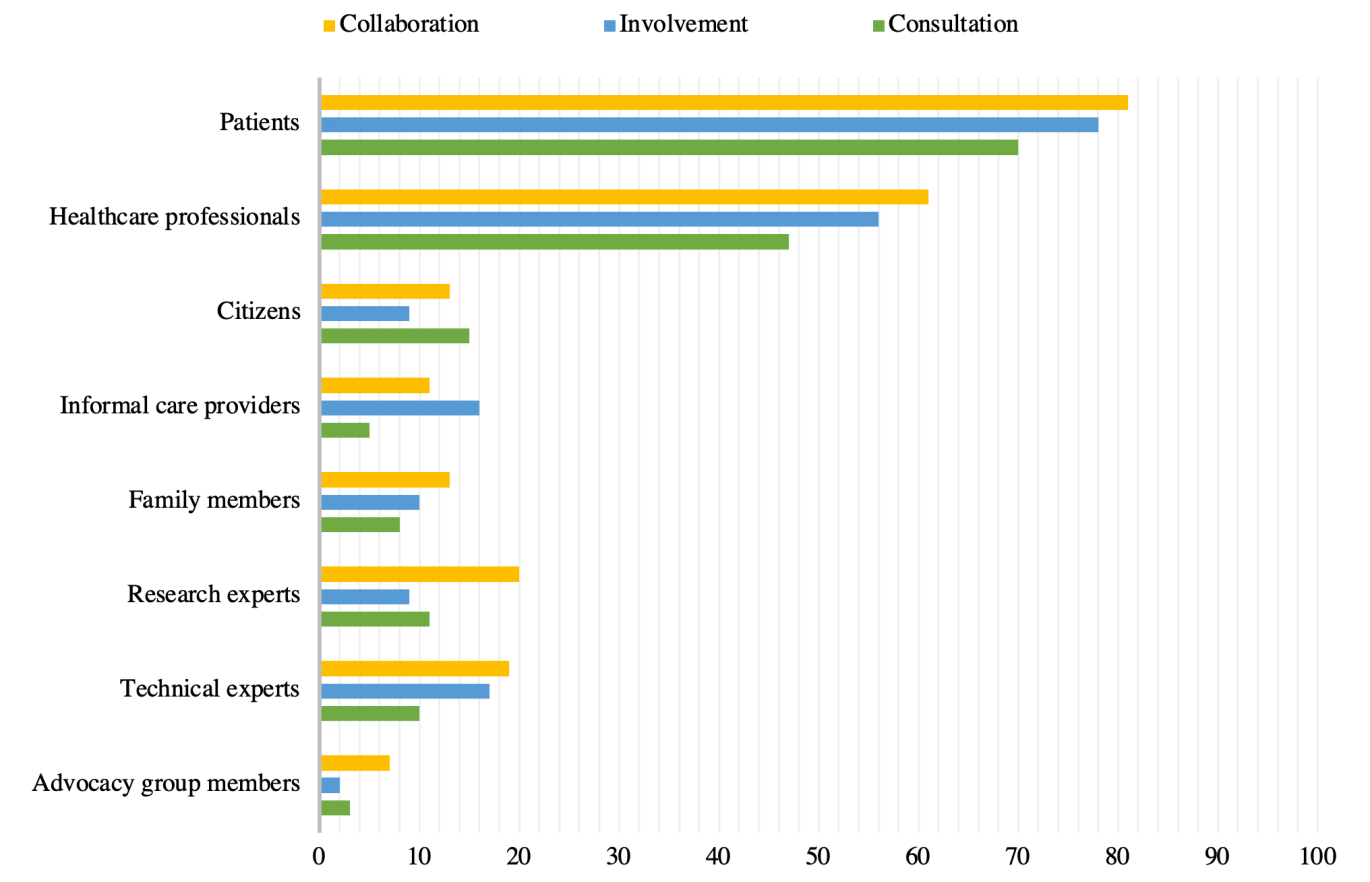
Types of Stakeholders Involved in Each Approach. Other, less frequently involved stakeholders are: student healthcare professionals, (n = 4 of all papers), managers, (n = 4), consultants (n = 2), marketing experts (n = 2), teachers (n = 1), hygiene instructors (n = 1), industry representatives (n = 1), inspectors (n=1), legal experts (n = 1), pharmacists (n = 1), policy makers (n = 1), and politicians (n = 1).

Figure 1 depicts the eight most-often involved types of stakeholders in the three approaches. Patients are involved in 81% of the collaborative approach papers, in 78% of the involvement approach papers, and in 70% of the consultation approach papers. Patients and healthcare professionals are most often included in all three participatory approaches.

 Not all included articles report data on stakeholder characteristics. In 39% of the papers, gender is not reported; 45% of the papers do not report information on age, and in 67% ethnicity is not reported. In the papers that do report gender, women are overrepresented in all participatory approaches: women make up 60% of the public involved in the development of gender-neutral devices.

###  Data Collection Methods Used in the Three Participatory Approaches

**Figure 2:**
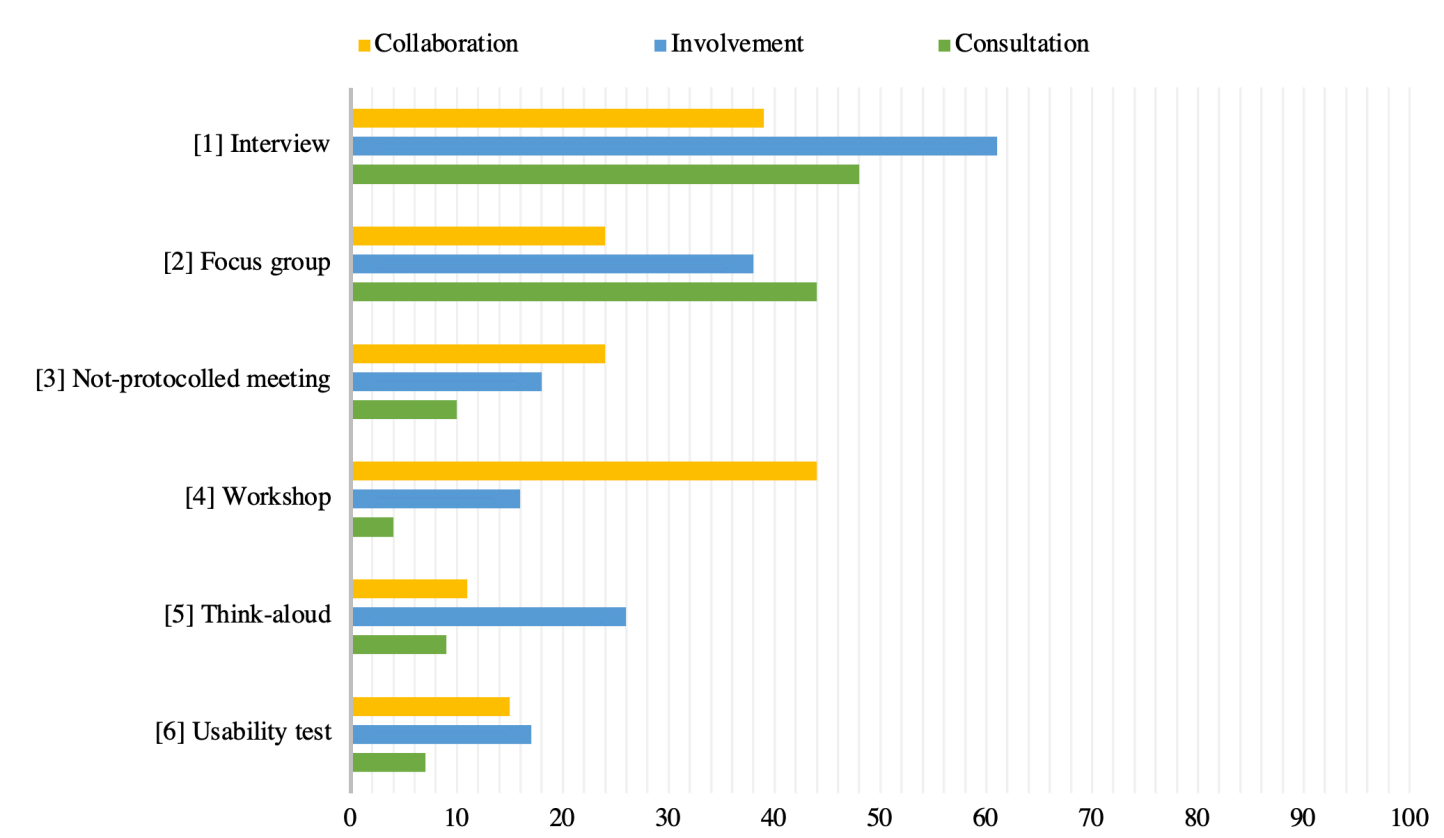
The Most Often Used Data Collection Methods in Each Approach. [1] A researcher asks questions to an individual with a (semi) structured question protocol. [2] When a researcher asks questions to a group of people with a (semi)structured protocol and the group is asked to talk over these questions. [3] Meetings with a researcher and an individual or group of people where no protocol is used. [4] Meetings with a researcher and a group of people where a protocol with questions and exercises is used. These exercises are often aimed at materially (with paper, pens, post-its) eg, persona, user flow or mock-ups. [5] Individuals are asked to use a mock-up or prototype and verbalise their thoughts while using it. Additional questions are sometimes asked. [6] Individuals are asked to use a mock-up or prototype and then give feedback, either open ended or protocolled. Other methods that are less frequently used are the [7] brainstorm, [8] heuristic evaluation [9] Delphi study, [10] forum, [11] nominal group technique, [12] observation, [13] questionnaire, [14] sketching individually, [15] diary writing experiences with device, [16] photovoice, where participants make pictures and explain why they made these pictures.

 In Figure 2, the six most often used data collection methods are presented for each approach. Interviews are most frequently employed in the involvement approach (61% of the papers). In the collaboration approach, workshops are most often used (44% of the papers), followed by interviews (39% of the papers). In the consultation approach, interviews are the most used data collection method (48% of the papers). Note that the consultation approach includes all individual data collection methods, so interviews and focus groups make up a large proportion of this category.

 It should be noted that some approaches, for example think-aloud and focus group, are listed in the consultation approach in Table as well as data collection method for all three approaches in Figure 2. This has two reasons. Fist, based on how the total research design was employed a paper was assigned to one of the three approaches. If a paper makes use of focus group as part a user-centred design, the paper is assigned to the involvement approach. If a focus group was performed to only inform specific choices, the paper is assigned to the consultation approach. Second, some individual papers present a data collection method as overarching participatory approach. Therefore, for example interviews are listed as research design as well as data collection method.

**Table:** Categorisation of the Participatory Approaches Based on the Levels of Participation of the Spectrum of Public Participation

**Overarching Research Approach and Level of Participation **	**Research Approach as Described in the Included Papers**
Collaboration (n = 75) *Equal collaboration between researchers and participants during the development process*	Co-design, Co-production, Co-creation, Collaboration (n = 36), Participatory design (n = 28), PAR, Action Research, Community-based participatory research (n = 7), Interdisciplinary design (n = 2), Inclusive design (n = 1), User-driven approach (n = 1)
Involvement (n = 98) *Participants are involved choices through the development process*	User-centred (n = 75), Staged/iterative (n = 6), User-experience/experience based (n = 5), Human-centred (n = 4), Person-based (n = 3), CeHRes roadmap (n = 2), Patient-centred (n = 2), Client-centred (n = 1)
Consultation (n = 104) *Participants are consulted to inform specific development choices*	Mixed-methods design (n = 21), Focus group study (n = 19), Interview study (n = 17), Qualitative research design (n = 9), Qualitative survey (n = 4), Delphi study (n = 2), Nominal group technique study (n = 1), Think-aloud study (n = 1), Design focussed study (n = 30)

Abbreviations: PAR, Participatory Action Research; CeHRes, Center for eHealth Research.

###  Topics Addressed in the Three Participatory Approaches

**Figure 3:**
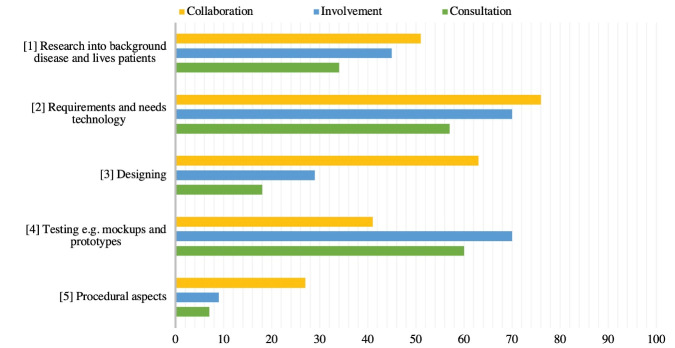
The Topics Addressed in the Approaches. [1] Includes research into the management of the disease, health behaviour, current care practices, current technology use, and user identification. [2] Contains topics about the functional requirements of a technology, needs that a technology should resolve, and contents a technology should have. [3] Includes making or influencing choices in the creation of preliminary frameworks, mock-ups, or prototypes. [4] Testing or reviewing the technology based on general impressions, usability, quality of content or functionality. [5] Consists of discussions about the method or procedure of the involvement, thoughts on participating, of discussing findings.

Figure 3 shows these five categories, and depicts how frequently these topics are discussed in each approach. All topics addressed in the papers are categorised into five overall categories. The first category is research into the disease and the lives of patients, such as experiences of stakeholders with the healthcare trajectory or disease, and previous device use. This topic is addressed in 51% of the collaboration approach papers, in 45% of the involvement approach papers, and in 34% of the consultation approach papers. Requirements and needs are addressed in 76% (collaboration), 36% (involvement) and 26% (consultation) of the papers. Designing is addressed in 63% of the collaboration approach papers, 29% of the involvement approach papers, and 18% of the consultation approach papers. Testing and reviewing the device is addressed in 41%, 70% and 59% in the involvement, collaboration, and consultation approach, respectively. Discussing procedural aspects encompasses a discussion about the aim of a specific engagement, discussing the findings, or reflections on the participation with the partakers. This topic is discussed in 27% of the collaboration approach papers, in 9% of the involvement approach papers, and in 8% of the consultation approach papers.

###  Challenges of the Three Participatory Approaches 

**Figure 4:**
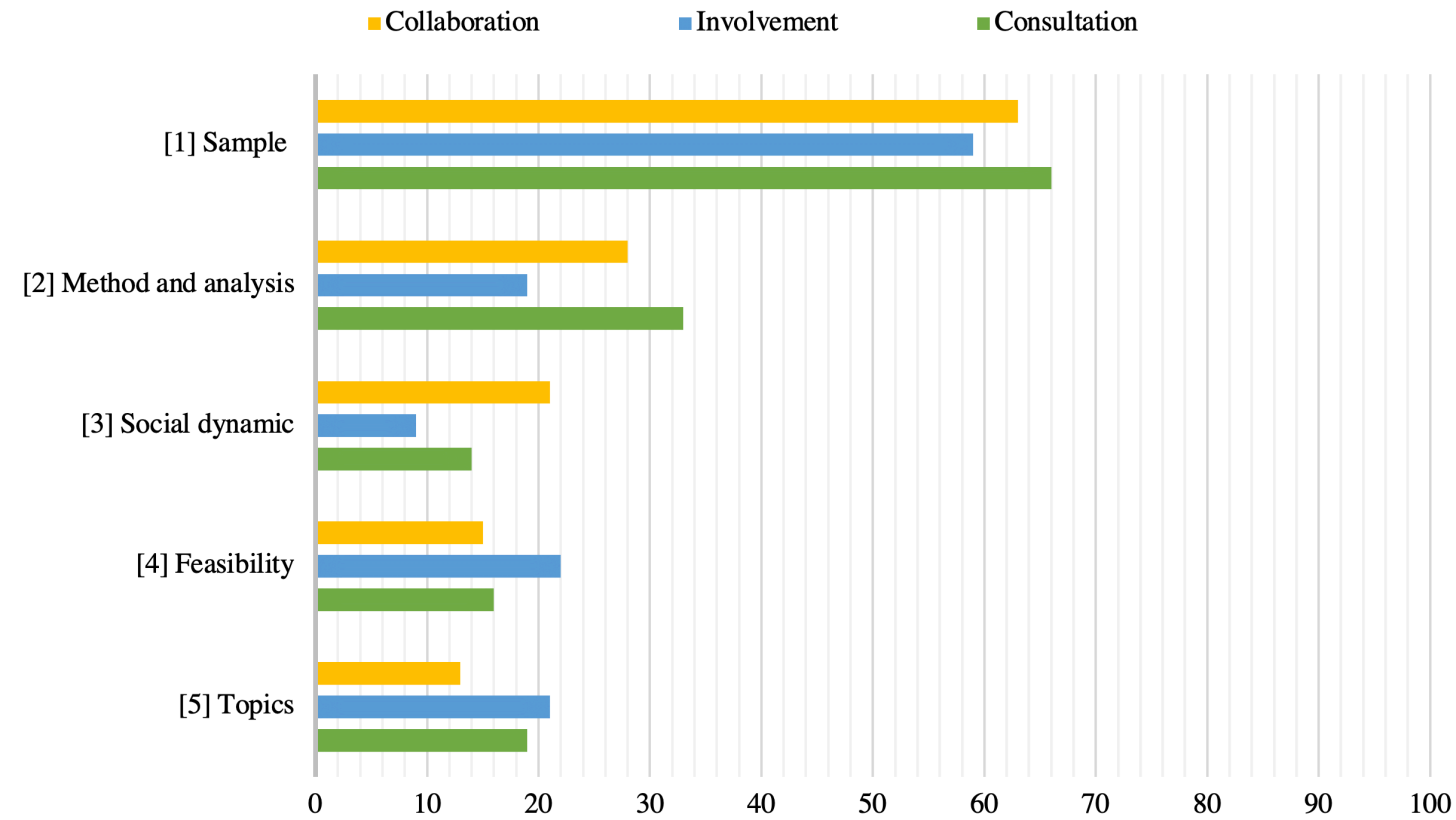
Five Types of Challenges That Occur in Each Approach. Explanation of identified challenges. [1] Sample: sampling limitations pertain the size of the sample and a not-representative or non-inclusive sample. [2] Method and analysis: includes issues on methodological aspects like not testing in a real-world environment, making changes in the method while data is collected, and performing the analysis by only one person. [3] Social dynamic: comprised of all limitations relating the dynamic between participants or participants and the researchers. [4] Feasibility: time, finance or other feasibility impediments. [5] Topics: not always all topics were addressed, like negative or ethical consequences of a device, adherence, or a limited number of functionalities.

 All challenges that occurred in the papers are grouped in five types of challenges. Figure 4 shows these 5 types of challenges, and depicts how frequently these challenges occurred within these approaches. The most reported challenge is related to sampling issues, which is reported in 63% of the collaboration approach papers, 59% of the involvement approach papers, and in 66% of the consultation approach papers. Sampling issues include a small sample size, a lack of representation of gender, racial diversity, living location, involved stakeholder types, or digital capabilities. Methodical and analysis issues, such as not testing in a real-world environment, or performing the analysis by one researcher, are most often identified in the collaboration (28%) and the consultation approach papers (33%). The social dynamic between participants or between the participants and moderators challenging the validity of the outcomes, is most frequently mentioned in the collaboration approach papers (21%). For example, some stakeholders can be timid in a group discussion, whereas others can be highly talkative. Feasibility issues such as limited financial resources to perform a study, were reported in all three approaches (respectively 15, 22, and 16%). The challenge to address all relevant topics is reported in 13% of the collaboration approach papers, 21% of the involvement approach papers and 19% of the consultation approach papers.

## Discussion

 This review shows that many participatory approaches are used in the development of medical devices that can be classified into three overarching approaches: the collaboration, the involvement, and the consultation approach. These approaches differ in the degree of power that is granted to stakeholders, but also have comparable characteristics. Patients and healthcare professionals appear to be most often involved in all three approaches, whereas workshops, interviews and focus groups were the most often used data collection methods in respectively the collaboration, involvement, and consultation approach. Topics addressed in all approaches are: the problem a device should help to solve, requirements of devices, design choices, testing of devices, and the procedure of involvement itself. In every approach, researchers experience challenges in selecting a representative sample of participants. The results also show that stakeholder characteristics such as gender, age, and ethnicity are infrequently reported. Participatory research is mainly conducted in Europe and America for the development of e-health technologies.

 We are the first to take a broad scope, including participatory approaches used in the development of all sorts of medical devices and reviewing literature published in and outside the medical field. Still, our results are in agreement with an earlier review by Moore et al^19^ who performed a systematic review on participatory approaches for specifically e-health technologies and focussed on the involvement of users rather than stakeholders in a broader sense. They found a comparable variety of data collection methods, ranging from focus groups to usability tests, and described five overall topics addressed: contextual inquiry (similar to what we described as ‘research into the disease and the lives of patients’), value specification (similar to requirements and needs), designing, operationalisation (or feasibility studies; in our results included under testing) and summative evaluation (similar to testing). So only the procedural aspects found in our study were not identified by Moore et al.^19^ An explanation could be that procedural aspects do not directly relate to the development of the device, but to the participatory process. Moore et al did not analyse which types of stakeholders were involved, the challenges that researcher experience when executing the approach, and did not account for different levels of participation. Our finding that e-health devices make up a large proportion of devices for which participatory approaches are used is in line with a finding in another review on user- and human centred design approaches for research and innovation, which also showed that much work is dedicated to e-health technologies.^21^

###  Strengths and Limitations

 This is the first review that focusses on how participatory approaches differ when they are applied in the development of medical devices. This review also offers the first comprehensive overview of all participatory approaches employed in medical device development, mapping out what approaches are used. Some potential limitations should be discussed. First, we analysed methodological characteristics and challenges as reported in the papers, rather than identified by ourselves. Some methodological characteristics might therefore be underreported in our overview, eg: unsystematic meetings that could consist of brief conversations, or stakeholder groups that researchers might not perceive as stakeholders, such as marketeers. This limit also applies to methodological challenges, because researchers might find specific challenges irrelevant to report. Second, the search largely consisted of keywords that we as researchers specified in multiple discussions. We could not exclusively use MeSH and Emtree terms – headings that cover a large diversity of different concepts that are synonymous - because concepts like ‘participation’ or ‘involvement’ do not have adequate MeSH terms in PubMed. As a consequence, possible relevant papers might have been missed in the search. We tried to account for this limitation by identifying terms in a variety of different articles when developing the search strategy, and by performing a broad scoping review we believe we were able to include all relevant concepts. Third, we did not focus on the impact of the reported participatory approaches on ensuing decision-making, eg, on how such approaches eventually influenced the designs of the devices. We consider this an important future line of research, yet beyond the scope of this review.

###  Implications

 The most important finding of this review is that the three participatory approaches do not differ much in terms of stakeholders involved, data collection methods, topics discussed, and challenges encountered, which implies that these approaches do not automatically come with specific methods to be used. This means that the level of participation, stakeholder selection, methods for data collection, and topics to be discussed involve independent choices researchers seeking to embed participatory approaches in medical device development have to make. Another implication is that it is relevant to analyse how participatory approaches are practically employed. Because it is seldomly analysed how participatory approaches are used in practice, there are many open questions. Questions one could raise are eg: what is the effect of using a workshop instead of a focus group? What are the benefits and caveats of including experts? And which strategies work best for involving a diverse and representative sample of stakeholders? Answers to these questions can help researchers to make well-informed choices when employing participatory research. Increased effort can be dedicated to the selection of stakeholders that fairly represent the stakeholder group of a device. The majority of papers reported that including a representative sample of stakeholders remains a challenge. Related to this point is that more work needs to be done to describe stakeholder characteristics, which appeared to be lacking in some papers. For readers it is hard to know who are consulted, and thus to know whose interests are taken in consideration in the development of medical device.

 In conclusion, participatory approaches reported in literature can be categorised in three overarching approaches: collaboration, involvement, and consultation. These approaches have similar methodological characteristics. We suggest that researchers interested in stakeholder participation should not focus on adopting a pre-determined approach. They can better flexibly determine the level of participation, types of stakeholders, methods, topics, and strategies to address challenges.

## Acknowledgements

 We wish to express our gratitude to On Ying Chan and Alice Tillema for their valuable help with composing the search strategy and Jonathan Jonker for his help with the data-extraction.

## Ethical issues

 Not applicable.

## Competing interests

 Authors declare that they have no competing interests.

## Authors’ contributions

 KW, RR, MR, and MT contributed to the conception and design. KW and MT contributed to the acquisition, analysis and interpretation of data. KW, RR, MR, and MT have been drafting the manuscript. MR has obtained funding. RR, MR, and RR have been supervising the project.

## Funding

 This work was supported by a Vici fund by the Nederlandse organisatie voor gezondheidsonderzoek en zorginnovatie (ZonMw) [grant number 91818617].

## Supplementary files


Supplementary file 1. Research Protocol.

Supplementary file 2. Search Strategy.

Supplementary file 3. Overview of Data Items.

Supplementary file 4. The PRISMA Flow Chart.

Supplementary file 5. Overview of Included Papers, Their Characteristics, and the reported Challenges Grouped in the Three Participatory Approaches.

Supplementary file 6. Literature List of Included Papers.
