# Supplementary material for: An Overview of Stakeholders, Methods, Topics, and Challenges in Participatory Approaches Used in the Development of Medical Devices: A Scoping Review
Source: Int J Health Policy Manag. 2022 Nov 5;12:6839. doi: 10.34172/ijhpm.2022.6839 (PMC10125077; doi:10.34172/ijhpm.2022.6839)
Supplement: Supplementary file 1 — Research Protocol. [file ijhpm-12-6839-s001.pdf]

Article title: An Overview of Stakeholders, Methods, Topics, and Challenges in Participatory Approaches Used in the Development of Medical Devices: A Scoping Review

Journal name: International Journal of Health Policy and Management (IJHPM)

Authors' information: Kas Woudstra[1]*, Rob Reuzel[2], Maroeska Rovers[2], Marcia Tummers[2]

[1]Department of Health Evidence and Operation Rooms, Radboud University Medical Center, Nijmegen, The Netherlands.

[2]Department of Health Evidence, Radboud University Medical Center, Nijmegen, The Netherlands.

(*Corresponding author: Kas.Woudstra@radboudumc.nl)

Supplementary file 1. Research Protocol

1. Protocol Review – Methods for stakeholder involvement in health sciences – a literature review and analysis on closure, trustworthiness, power and applicability

3-4. Introduction

The past decade is marked by a transition from doing research and innovation and making policy *for* society, to doing research and innovation *with* society. The inclusion of stakeholders in health sciences is widely regarded as important within the research community (Gagnon et al., 2011; Assasi et al., 2014). Even though the urge of stakeholder inclusion is broadly felt, an overview of existing inclusion methods does not exist. Neither are these methods evaluated on characteristics such as applicability and power. For users of stakeholder inclusion methods in health sciences, ranging from engineers to scientists and policy makers, it us currently hard to know which methods are at hand, even harder to grasp what the distinct characteristics of these methods are, and not possible to compare these methods on their characteristics. Hence the research question is as follows:

Research question
What are the characteristics and effects of stakeholder involvement methods in the development of medical technology?

Methods

6. Eligibility criteria

Included studies:

- Present original study data
- Perform a method of stakeholder involvement
- Concern medical technology

- In the development phase or the technology
- In the domain of health care
- Are written in English
- Are published in 2015, 2016, 2017, 2018 or 2019

7. Information sources

Pubmed, Embase and Web of Science are the databases in which the studies will be searched for.

8. Search strategy

We will use synonyms of (1) technology, (2) designing, (3) stakeholders, (4) involvement, (5) method, in the domain of (6) healthcare. Included studies will be screened on their reference lists to assess whether the literature search identified all relevant papers in accordance with the backwards-snowballing method.

Technology	Design	User	Engage	Method	Health
Prototype	Development	Stakeholder	Participate	Methods	Healthcare
Device	Innovate	Patient	Involve		
Digital		Professional	Include		
App		Community	Collaborate		
		Public			

9. Study records

Selection process

Duplicates will be removed. One authors will identify relevant studies by screening title and abstracts. These relevant studies will be fully screened based on the eligibility criteria. This again will be done by both the two authors, independently. In case of disagreement upon inclusion, the third author will be consulted.

10. Data management and collection

Via website [www.rayyan.qcri.org.](http://www.rayyan.qcri.org) the titles and abstracts will be collected and screened.

11. Data items

On the following variables information will be collected:

1. Names of authors
2. Year of publication
3. Country of study setting
4. Device as named in article
5. Name of research approach
6. Description of research approach
7. Types of stakeholders that are engaged during development
8. Percentage of female participants involved in each approach (for gender-neutral devices)
9. Age of participants
10. Ethnicity of participants

11. Data-collection methods
12. Topics that are discussed with stakeholders
13. Challenges that occur by applying the participatory research approaches as described by the researchers

Risk of bias

Only articles in English are screened. Hence there will be an Anglo-European bias in the selection.

Current search:

Pubmed
(("technology"[MeSH Terms] OR Technology[tiab] OR technologies[tiab] OR technological[tiab] OR Prototype[tiab] OR Prototypes[tiab] OR Prototyping[tiab] OR Device[tiab] OR Devices[tiab] OR Digital[tiab] OR app[tiab] OR apps[tiab]) AND ("equipment design"[Mesh] OR design[tiab] OR designs[tiab] OR designing[tiab] OR designed[tiab] OR designer[tiab] OR designers[tiab] OR development[tiab] OR develop[tiab] OR develops[tiab] OR developing[tiab] OR developed[tiab] OR developer[tiab] OR developers[tiab] OR Innovation[tiab] OR Innovations[tiab] OR Innovate[tiab] OR Innovated[tiab] OR Innovating[tiab] OR innovator[tiab] OR innovators[tiab]) AND (User[tiab] OR Users[tiab] OR Stakeholder[tiab] OR Stakeholders[tiab] OR Patient[tiab] OR Patients[tiab] OR Participant[tiab] OR Participants[tiab] OR Professional[tiab] OR Professionals[tiab] OR Community[tiab] OR Communities[tiab] OR Public[tiab] OR Publics[tiab] OR citizen[tiab] OR citizens[tiab] OR expert[tiab] OR experts[tiab]) AND ("community participation"[mesh] OR "stakeholder participation"[mesh] OR Engage[tiab] OR Engaged[tiab] OR Engagement[tiab] OR Engages[tiab] OR Engaging[tiab] OR Participate[tiab] OR Participated[tiab] OR Participation[tiab] OR Participates[tiab] OR Participating[tiab] OR Involve[tiab] OR Involved[tiab] OR Involvement[tiab] OR Involves[tiab] OR Involvements[tiab] OR Involving[tiab] OR Include[tiab] OR Included[tiab] OR Includes[tiab] OR Including[tiab] OR Inclusion[tiab] OR Collaboration[tiab] OR Collaborate[tiab] OR Collaborated[tiab] OR Collaborates[tiab] OR Collaborating[tiab]) AND ("methods"[MeSH Terms] OR Method[tiab] OR Methods[tiab]) AND (Health[tiab] OR Healthcare[tiab] OR "health care"[tiab])) AND ("2014/07/01"[PDat] : "2019/06/30"[PDat])
**Web of Science**
((technology OR technologies OR technological OR Prototype OR Prototypes OR Prototyping OR Device OR Devices OR Digital OR app OR apps) AND (design OR designs OR designing OR designed OR designer OR designers OR development OR develop OR develops OR developing OR developed OR developer OR developers OR Innovation OR Innovations OR Innovate OR Innovated OR Innovating OR innovator OR innovators) AND (User OR Users OR Stakeholder OR Stakeholders OR Patient OR Patients OR Participant OR Participants OR Professional OR Professionals OR Community OR Communities OR Public OR Publics OR citizen OR citizens OR expert OR experts) AND (Engage OR Engaged OR Engagement OR Engages OR Engaging OR Participate OR Participated OR Participation OR Participates OR Participating OR Involve OR Involved OR Involvement OR Involves OR Involvements OR Involving OR Include OR Included OR Includes OR Including OR Inclusion OR Collaboration OR Collaborate OR Collaborated OR Collaborates OR Collaborating) AND (Method OR Methods) AND (Health OR Healthcare))
**Embase**

(exp technology/ or Technology.ti,ab,kf. or technologies.ti,ab,kf. or technological.ti,ab,kf. or Prototype.ti,ab,kf. or Prototypes.ti,ab,kf. or Prototyping.ti,ab,kf. or Device.ti,ab,kf. or Devices.ti,ab,kf. or Digital.ti,ab,kf. or app.ti,ab,kf. or apps.ti,ab,kf.) and (exp "equipment design"/ or design.ti,ab,kf. or designs.ti,ab,kf. or designing.ti,ab,kf. or designed.ti,ab,kf. or designer.ti,ab,kf. or designers.ti,ab,kf. or development.ti,ab,kf. or develop.ti,ab,kf. or develops.ti,ab,kf. or developing.ti,ab,kf. or developed.ti,ab,kf. or developer.ti,ab,kf. or developers.ti,ab,kf. or Innovation.ti,ab,kf. or Innovations.ti,ab,kf. or Innovate.ti,ab,kf. or Innovated.ti,ab,kf. or Innovating.ti,ab,kf. or innovator.ti,ab,kf. or innovators.ti,ab,kf.) and (User or Users or Stakeholder or Stakeholders or Patient or Patients or Participant or Participants or Professional or Professionals or Community or Communities or Public or Publics or citizen or citizens or expert or experts).ti,ab,kf. and (exp "community participation"/ or exp "stakeholder participation"/ or Engage.ti,ab,kf. or Engaged.ti,ab,kf. or Engagement.ti,ab,kf. or Engages.ti,ab,kf. or Engaging.ti,ab,kf. or Participate.ti,ab,kf. or Participated.ti,ab,kf. or Participation.ti,ab,kf. or Participates.ti,ab,kf. or Participating.ti,ab,kf. or Involve.ti,ab,kf. or Involved.ti,ab,kf. or Involvement.ti,ab,kf. or Involves.ti,ab,kf. or Involvements.ti,ab,kf. or Involving.ti,ab,kf. or Include.ti,ab,kf. or Included.ti,ab,kf. or Includes.ti,ab,kf. or Including.ti,ab,kf. or Inclusion.ti,ab,kf. or Collaboration.ti,ab,kf. or Collaborate.ti,ab,kf. or Collaborated.ti,ab,kf. or Collaborates.ti,ab,kf. or Collaborating.ti,ab,kf.) and (exp methods/ or Method.ti,ab,kf. or Methods.ti,ab,kf.) and (Health or Healthcare or "health care").ti,ab,kf.
