# Supplementary material for: An Overview of Stakeholders, Methods, Topics, and Challenges in Participatory Approaches Used in the Development of Medical Devices: A Scoping Review
Source: Int J Health Policy Manag. 2022 Nov 5;12:6839. doi: 10.34172/ijhpm.2022.6839 (PMC10125077; doi:10.34172/ijhpm.2022.6839)
Supplement: Supplementary file 2 — Search Strategy. [file ijhpm-12-6839-s002.pdf]

Article title: An Overview of Stakeholders, Methods, Topics, and Challenges in Participatory Approaches Used in the Development of Medical Devices: A Scoping Review

Journal name: International Journal of Health Policy and Management (IJHPM)

Authors' information: Kas Woudstra[1]*, Rob Reuzel[2], Maroeska Rovers[2], Marcia Tummers[2]

[1]Department of Health Evidence and Operation Rooms, Radboud University Medical Center, Nijmegen, The Netherlands.

[2]Department of Health Evidence, Radboud University Medical Center, Nijmegen, The Netherlands.

(*Corresponding author: Kas.Woudstra@radboudumc.nl)

Supplementary file 2. Research Protocol

The search was conducted on March the 31th, 2022

**Pubmed**
(("technology"[MeSH Terms] OR Technology[tiab] OR technologies[tiab] OR technological[tiab] OR Prototype[tiab] OR Prototypes[tiab] OR Prototyping[tiab] OR Device[tiab] OR Devices[tiab] OR Digital[tiab] OR app[tiab] OR apps[tiab]) AND ("equipment design"[Mesh] OR design[tiab] OR designs[tiab] OR designing[tiab] OR designed[tiab] OR designer[tiab] OR designers[tiab] OR development[tiab] OR develop[tiab] OR develops[tiab] OR developing[tiab] OR developed[tiab] OR developer[tiab] OR developers[tiab] OR Innovation[tiab] OR Innovations[tiab] OR Innovate[tiab] OR Innovated[tiab] OR Innovating[tiab] OR innovator[tiab] OR innovators[tiab]) AND (User[tiab] OR Users[tiab] OR Stakeholder[tiab] OR Stakeholders[tiab] OR Patient[tiab] OR Patients[tiab] OR Participant[tiab] OR Participants[tiab] OR Professional[tiab] OR Professionals[tiab] OR Community[tiab] OR Communities[tiab] OR Public[tiab] OR Publics[tiab] OR citizen[tiab] OR citizens[tiab] OR expert[tiab] OR experts[tiab]) AND ("community participation"[mesh] OR "stakeholder participation"[mesh] OR Engage[tiab] OR Engaged[tiab] OR Engagement[tiab] OR Engages[tiab] OR Engaging[tiab] OR Participate[tiab] OR Participated[tiab] OR Participation[tiab] OR Participates[tiab] OR Participating[tiab] OR Involve[tiab] OR Involved[tiab] OR Involvement[tiab] OR Involves[tiab] OR Involvements[tiab] OR Involving[tiab] OR Include[tiab] OR Included[tiab] OR Includes[tiab] OR Including[tiab] OR Inclusion[tiab] OR Collaboration[tiab] OR Collaborate[tiab] OR Collaborated[tiab] OR Collaborates[tiab] OR Collaborating[tiab]) AND ("methods"[MeSH Terms] OR Method[tiab] OR Methods[tiab]) AND (Health[tiab] OR Healthcare[tiab] OR "health care"[tiab])) AND ("2014/07/01"[PDat] : "2019/06/30"[PDat])
Hits: 7794
**Web of Science**
((technology OR technologies OR technological OR Prototype OR Prototypes OR Prototyping OR Device OR Devices OR Digital OR app OR apps) AND (design OR designs OR designing OR designed OR designer OR designers OR development OR develop OR develops OR developing OR developed OR developer OR developers OR Innovation OR Innovations OR Innovate OR Innovated OR Innovating OR innovator OR innovators) AND (User OR Users OR Stakeholder OR Stakeholders OR Patient OR Patients OR Participant OR Participants OR Professional OR Professionals OR Community OR Communities OR Public OR Publics OR citizen OR citizens OR expert OR experts) AND (Engage OR Engaged OR Engagement OR Engages OR Engaging OR Participate OR Participated OR Participation OR Participates OR Participating OR Involve OR Involved OR Involvement OR Involves OR Involvements OR Involving OR Include OR Included OR Includes OR Including OR Inclusion OR Collaboration OR Collaborate OR Collaborated OR Collaborates OR Collaborating) AND (Method OR Methods) AND (Health OR Healthcare))
Hits: 8004
**Embase**

(exp technology/ or Technology.ti,ab,kf. or technologies.ti,ab,kf. or technological.ti,ab,kf. or Prototype.ti,ab,kf. or Prototypes.ti,ab,kf. or Prototyping.ti,ab,kf. or Device.ti,ab,kf. or Devices.ti,ab,kf. or Digital.ti,ab,kf. or app.ti,ab,kf. or apps.ti,ab,kf.) and (exp "equipment design"/ or design.ti,ab,kf. or designs.ti,ab,kf. or designing.ti,ab,kf. or designed.ti,ab,kf. or designer.ti,ab,kf. or designers.ti,ab,kf. or development.ti,ab,kf. or develop.ti,ab,kf. or develops.ti,ab,kf. or developing.ti,ab,kf. or developed.ti,ab,kf. or developer.ti,ab,kf. or developers.ti,ab,kf. or Innovation.ti,ab,kf. or Innovations.ti,ab,kf. or Innovate.ti,ab,kf. or Innovated.ti,ab,kf. or Innovating.ti,ab,kf. or innovator.ti,ab,kf. or innovators.ti,ab,kf.) and (User or Users or Stakeholder or Stakeholders or Patient or Patients or Participant or Participants or Professional or Professionals or Community or Communities or Public or Publics or citizen or citizens or expert or experts).ti,ab,kf. and (exp "community participation"/ or exp "stakeholder participation"/ or Engage.ti,ab,kf. or Engaged.ti,ab,kf. or Engagement.ti,ab,kf. or Engages.ti,ab,kf. or Engaging.ti,ab,kf. or Participate.ti,ab,kf. or Participated.ti,ab,kf. or Participation.ti,ab,kf. or Participates.ti,ab,kf. or Participating.ti,ab,kf. or Involve.ti,ab,kf. or Involved.ti,ab,kf. or Involvement.ti,ab,kf. or Involves.ti,ab,kf. or Involvements.ti,ab,kf. or Involving.ti,ab,kf. or Include.ti,ab,kf. or Included.ti,ab,kf. or Includes.ti,ab,kf. or Including.ti,ab,kf. or Inclusion.ti,ab,kf. or Collaboration.ti,ab,kf. or Collaborate.ti,ab,kf. or Collaborated.ti,ab,kf. or Collaborates.ti,ab,kf. or Collaborating.ti,ab,kf.) and (exp methods/ or Method.ti,ab,kf. or Methods.ti,ab,kf.) and (Health or Healthcare or "health care").ti,ab,kf.
limit 1 to dc=20140701-20190630
limit 2 to conference abstract
2 not 3
Hits: 9178
**Hits total, deduplicated: 24976**
