# Supplementary material for: An Overview of Stakeholders, Methods, Topics, and Challenges in Participatory Approaches Used in the Development of Medical Devices: A Scoping Review
Source: Int J Health Policy Manag. 2022 Nov 5;12:6839. doi: 10.34172/ijhpm.2022.6839 (PMC10125077; doi:10.34172/ijhpm.2022.6839)
Supplement: Supplementary file 3 — Overview of Data Items. [file ijhpm-12-6839-s003.pdf]

Article title: An Overview of Stakeholders, Methods, Topics, and Challenges in Participatory Approaches Used in the Development of Medical Devices: A Scoping Review

Journal name: International Journal of Health Policy and Management (IJHPM)

Authors' information: Kas Woudstra[1]*, Rob Reuzel[2], Maroeska Rovers[2], Marcia Tummers[2]

[1]Department of Health Evidence and Operation Rooms, Radboud University Medical Center, Nijmegen, The Netherlands.

[2]Department of Health Evidence, Radboud University Medical Center, Nijmegen, The Netherlands.

(*Corresponding author: Kas.Woudstra@radboudumc.nl)

Supplementary file 3. Overview of Data Items

1. Names of authors
2. Year of publication
3. Country of study setting
4. Device as named in article
5. Name of research approach
6. Description of research approach
7. Types of stakeholders that are engaged during development
8. Percentage of female participants involved in each approach (for gender-neutral devices)
9. Age of participants
10. Ethnicity of participants
11. Data-collection methods
12. Topics that are discussed with stakeholders
13. Challenges that occur by applying the participatory research approaches as described by the researchers
