# Supplementary material for: An Overview of Stakeholders, Methods, Topics, and Challenges in Participatory Approaches Used in the Development of Medical Devices: A Scoping Review
Source: Int J Health Policy Manag. 2022 Nov 5;12:6839. doi: 10.34172/ijhpm.2022.6839 (PMC10125077; doi:10.34172/ijhpm.2022.6839)
Supplement: Supplementary file 5 — Overview of Included Papers, Their Characteristics, and the reported Challenges Grouped in the Three Participatory Approaches. [file ijhpm-12-6839-s005.pdf]

Article title: An Overview of Stakeholders, Methods, Topics, and Challenges in Participatory Approaches Used in the Development of Medical Devices: A Scoping Review

Journal name: International Journal of Health Policy and Management (IJHPM)

Authors' information: Kas Woudstra[1]*, Rob Reuzel[2], Maroeska Rovers[2], Marcia Tummers[2]

[1]Department of Health Evidence and Operation Rooms, Radboud University Medical Center, Nijmegen, The Netherlands.

[2]Department of Health Evidence, Radboud University Medical Center, Nijmegen, The Netherlands.

(*Corresponding author: Kas.Woudstra@radboudumc.nl)

Supplementary file 5. Overview of Included Papers, Their Characteristics, and the reported Challenges Grouped in the Three Participatory Approaches

Table D1: Papers grouped in the collaboration approach

Nr	Authors	Year	Country	Technology	Approach	Stakeholders	Data collection methods	Topics	Challenges
1	Abrines Jaume et al.	2015	UK	An interactive application presented on a touch screen device that used animation to collect information on the health status of children	Collaboration	Patients, citizens	Interactive group session (woekshop)	Background, requirements, designing, procedure	Sample, social dynamic
2	Allin et al.	2018	Canada	Internet self-management program for spinal cord injury	Participatory design	Patients, HCPs, technical experts	Workshop, not-protocolled meeting	Background, requirements, designing, procedure	Sample
3	Baldereschi et al.	2016	Italy	A web app, named ICTUS3R, which refers to the three main messages: recognize stroke signs—react immediately and properly—reduce stroke risk	Collaboration	HCPs, citizens, technical experts, advocacy group members	Not-protocolled meeting, heuristic evaluation	Background, requirements	Not reported
4	Baskerville et al.	2018	Canada	Smartphone app for smoking cessation	Spiral technology action research	Patients, research experts, technical experts	Focus group, workshop	Background, requirements, designing	Not reported
5	Bjerkan et al.	2015	Norway	A web-based plan for integrated care	Participatory design	Patients, relatives	Interview	Designing, testing	Sample, method and analysis, topic
6	Boman et al.	2016	Sweden	All-in-one device that is based on ICT for persons with cognitive impairment after acquired brain injury and persons with mild and moderate dementia	Inclusive design method	Patients, relatives, HCPs	Interview, focus group	Requirements, designing	Sample, feasibility
7	Bourazeri et al.	2018	UK	A smart home toolset for persons living with early-stage dementia or Parkinson's to plan, monitor and self-manage his or her life	PERCEPT (PERrsona-CEntred Participatory Technology) approach	Patients, informal care givers	Workshop	Designing	Sample
8	Ceasar et al.	2019	USA	mHealth-enabled physical activity and cardiovascular health intervention (Step It Up)	Community-based participatory research	Patients, citizens	Focus group	Testing	Sample, feasibility, topics
9	Chhoun et al.	2019	Cambodia	mHealth intervention for female entertainment workers in Cambodia HIV as well as other sexual and reproductive health (SRH) outcomes	Iterative, participatory approach	Citizens	Interview, focus group, workshop	Procedure	Sample, social dynamic, topic
10	Cnossen et al.	2015	Netherlands	Web-based self-care program supporting early rehabilitation among patients after total laryngectomy	Participatory design approach	Patients, relatives, HCPs	Focus group, think-aloud, usability test	Background, requirements, testing, procedure	Sample
11	Cordova et al.	2015	USA	mHealth HIV/STI and drug abuse preventive intervention for primary care	Community-based participatory research	Patients	Focus group, interview	Requirements, designing, procedure	Sample
12	Crosby et al.	2017	USA	iManage: A self-management app co-designed by adolescents with sickle cell disease	Co-design	Patients	Interview, heuristic evaluation	Requirements, designing, testing	Sample, method and analysis
13	Dack et al.	2019	UK	A digital self-management intervention for adults with type 2 diabetes	Participatory design	Patients, HCPs	Interview, focus group	Testing	Sample, feasibility
14	Davis et al.	2018	Australia	An asthma s smartphone app for improving self-management, motivation and confidence in young people with asthma.	Patient-driven participatory design approach	Patients	Workshop, heuristic evaluation, observation	Designing, testing	Sample
15	de Souza et al.	2017	UK	A mobile application which provides relevant information for outpatient clinic attendees and data capture for clinicians	Co-design	Patients	Focus group, heuristic evaluation	Background, requirements, designing, testing, procedure	Social dynamic

Nr	Authors	Year	Country	Technology	Approach	Stakeholders	Data collection methods	Topics	Challenges
16	Duval et al.	2018	USA	A novel speech therapy game	Co-creation	HCPs	Interviews	Requirements, testing	Not reported
17	Dykes et al.	2014	USA	Web-based patient centred toolkit (PCTK)prototype to improve access to health information and to engage hospitalized patients and caregivers in the plan of care.	Iterative participatory design approach	Patients, relatives	Focus group, interview, not-protocolled meeting	Background, requirements, designing, testing, procedure	Sample
18	Easton et al.	2019	UK	Virtual agent to support individuals living with physical and mental comorbidities	Co-design and acceptability testing	Patients, informal care givers, HCPs	Workshop, usability test	Requirements, testing	Sample, social dynamic, topics
19	Elbaek et al.	2018	Denmark	e-Wall, a digital interactive gameified wall for movement	Collaboration / mixed methods approach	Patients, HCPs	Not-protocolled meeting, think-aloud	Background, requirements, testing	Method and analysis
20	Elsbernd et al.	2018	Denmark	Smartphone app for adolescents and young adults with cancer	Co-creation process	Patients	Workshop	Requirements	Sample, method and analysis
21	Ettinger et al.	2016	South Africa	ClinicalGuide, a mHealth app	User driven approach	HCPs, informal care givers, research experts, technical experts	Unsystematic meeting	Background, requirements, designing	Sample, method and analysis
22	Feldner et al.	2019	USA	Mobility device	Co-production	Patients, relatives	Photovoice	Background, requirements, designing, procedure	Sample, method and analysis, social dynamic, feasibility
23	Gardsten et al.	2017	Sweden	Information and communication technology (ICT) self-management service	Participatory design	Patients, HCPs, research experts	Focus group	Background	Sample
24	Goncu-Berk and Topcuoglu	2017	Turkey	a Smart Glove for Rheumatoid Arthritis	Iterative and multifaceted design process	Patients, HCPs	Interview, heuristic evaluation	Background, requirements, designing, testing	Not reported
25	Gordon et al.	2016	USA	eHealth solutions for women from vulnerable populations with perinatal depression	Participatory design group	Patients, HCPs, research experts, technical experts, managers	Workshop	Requirements, testing	Not reported
26	Grainger et al.	2017	New Zealand	RAConnect, a patient-held app for RA monitoring and communication with rheumatology care providers	Co-design	Patients, HCPs	Interview, heuristic evaluation	Background, requirements, designing	Not reported
27	Groussard et al.	2018	Canada	An assistive device, SAMI (Services Assistance Mobile and Intelligent), to improve the social participation of people with tuberculosis	Participatory design approach	Patients, HCPs	Interview, focus group, questionnaire	Background, requirements	Method and analysis, topics
28	Guedon et al.	2015	The Netherlands	Real-Time Location Systems in the Operating Room	Participatory approach	HCPs	Interview	Background, requirements, designing	Method and analysis
29	Hauffman et al.	2017	Sweden	Nurse-Led Internet-Based Learning and Self-care Program for Cancer Patients With Symptoms of Anxiety and Depression	Interdisciplinary multiprofessional working group	Patients, HCPs	Focus group	Background, requirements, testing	Sample
30	Ho et al.	2015	Canada	Metabolic diet app suite for inborn errors of amino acid metabolism	Collaboration	HCPs, citizens, advocacy group members, student HCPs	Workshop, think aloud, questionnaire	Procedure	Method and analysis
31	Hochstenbach et al.	2017	Netherlands	eHealth nursing intervention: self-management support for outpatients with cancer pain	Co-creative method	Patients, HCPs, research experts, technical experts	Interview, brainstorm, not-protocolled meeting, questionnaire	Background, requirements, testing	Method and analysis, feasibility, topics
32	Hodson et al.	2019	Canada	Mellow: a self- help holistic crisis planning mobile application by youth, for youth	Co-design	Patients, HCPs, policy makers	Interview, workshop	Background, requirements, designing, testing	Feasibility
33	Holm et al.	2017	Denmark	A clinical telehealth service for neonatal homecare	Collaboration	Patients, HCPs	Workshop	Background, requirements, procedure	Sample
34	Islind et al	2019	Sweden	An information portal (front-end of the platform) for the patients to access information to feed their self-care process	Co-designing with boundary objects	Patients, HCPs, research experts	Workshop	Background, requirements, designing, testing, procedure	Not reported
35	Jaensson et al.	2015	Sweden	Recovery Assessments by Phone Points (RAPP): A Mobile Phone App for Postoperative Recovery Monitoring and Assessment	Interdisciplinary task force and needs and app evaluation	HCPs, research experts	Unsystematic meeting	Background, requirements, designing	Social dynamic, topics
36	Jamin et al.	2017	Netherlands	VENSTER, an interactive artwork for nursing home residents with dementia	Cocreation	Patients, informal care givers, HCPs, HC manager	Workshop, think-aloud	Requirements, testing	Sample, social dynamic
37	Joensson et al.	2019	Denmark	A cardiac telerehabilitation web portal, called the ‘HeartPortal’, for use among heart failure (HF) patients	Participatory design	Patients, HCPs	Workshop	Requirements, designing	Sample

Nr	Authors	Year	Country	Technology	Approach	Stakeholders	Data collection methods	Topics	Challenges
38	Kildea et al.	2019	Canada	A Person-Centered Patient Portal	Stakeholder co-design approach	HCPs, technical experts	Unsystematic meeting	Background, designing, testing, procedure	Sample, method and analysis
39	Kim et al.	2015	USA	iN Touch mobile self-management program for overweight/obesity	Participatory design approach	Patients	Interview, unsystematic meeting, questionnaire	Requirements	Sample
40	Lee et al.	2017	USA	A social robot for adults with depression	Participatory design	Patients, HCPs	Interview	Designing	Sampling, social dynamics
41	Leorin et al.	2019	Italy	eHealth technologies for people with dementia	Co-design	Research experts	Workshop	Designing	Sample, social dynamic
42	Lipson-Smith et al.	2019	Australia	Audio-recording mobile app for people with cancer: the Secondears App	Co-design and stakeholder engagement	Patients, HCPs, technical experts, legal experts, advocacy group members, managers	Not-protocolled meeting, workshop	Background, requirements, designing, testing	Sample
43	Marent et al.	2018	UK, Portugal, Belgium, Kroatia	mHealth platform for HIV Care	Co-design process	Patients, HCPs	Workshop, interview	Background, requirements	Sample, method and analysis
44	Marent et al.	2018	UK	mHealth platform to support follow-up and self-management in HIV	Co-design	Patients, HCPs, technical experts	Interview, workshop	Requirements, testing, procedure	Not reported
45	Michalak et al.	2019	Canada	Web-based bipolar wellness centre	Community-based participatory research	Patients	Interview	Background, procedure	Sample
46	Nagele et al.	2018	Panama, Columbia, USA, Croatia, Denmark	Self-adiministered "Localized Morphine medicine" device	Participatory Design and Design Fiction	Patients, HCPs	Workshop, observation	Requirements, testing	Social dynamics
47	Newby et al.	2019	UK	Digital intervention to increase condom use amongst those selftesting for chlamydia (Wrapped)	Co-design methods	Patients, citizens, HCPs, research experts, technical experts, artists, advocacy group members	Not-protocolled meeting, workshop, think-aloud	Requirements, designing, testing	Not reported
48	Noergaard et al.	2017	Denmark	Web-based health care intervention for patients with heart disease	Participatory design study	Patients, HCPs	Workshop	Requirements, testing, procedure	Sample, social dynamic
49	O'Brien et al.	2016	UK	Web-based intervention for people in the retirement transition	Iterative co-design process	Citizens, HCPs, research experts	Workshop, not-protocolled meeting	Requirements, designing, testing	Sample, feasibility
50	Ogrin et al.	2018	Australia	A diabetes foot app	Co-design	Patients, relatives, technical experts	Workshop	Requirements, designing, testing	Sample, social dynamic, feasibility
51	Ortiz-Fernandez et al.	2018	Spain	"Decision SupporT and Self- Management System for StRoke SurvivoRs" (STARR) for self- management and reduction of recurrent strok	Participatory action research	Patients	Interview	Requirements, designing, procedure	Sample, feasibility
52	Ospina-Pinillos et al.	2018	Australia	Mental health eclinic to improve access to and quality of mental health care for young people	Participatory design methodologies	Patients, HCPs	Workshop, think-aloud	Background, requirements, designing, testing	Sample
53	Page	2018	USA	Cochlear implant (CI) systems	Co-design 'Speculative Co-design' approach	Patients, HCPs	Workshop	Requirements, designing, procedure	Not reported
54	Panek et al.	2017	Austria	A new type of ICT enhanced modular toilet system which shall be able to support autonomy, dignity and safety of older persons living at home	Participatory Design	Patients, HCPs, informal care givers	Interview, focus group	Requirements, designing	Not reported
55	Qin et al.	2017	China	An ICIS a critical care information system in a Chinese hospital	Participative design process	HCPs	Unsystematic meeting, heuristic evaluation	Background, requirements	Sample
56	Rasmussen et al.	2015	USA	The Applicator: an easy to operate pump-action lever providing consistently accurate dose delivery	Collaborative Approach	Patients, HCPs	Interview, workshop, photovoice	Background, requirements, testing, procedure	Not reported
57	Reaple et al.	2019	UK	Virtual world with young people to deliver social cognition therapy in early psychosis	Co-design	Citizens	Workshop	Requirements, designing, testing	Feasibility
58	Redwood et al	2017	UK	New universal assessment tool named "My Family Profile" for use within Northamptonshire, United Kingdom, from pregnancy until a child reaches 2/2.5 years of age	Co-design	Parents	Not-protocolled meeting, focus group, Interview, usability test	Background, requirements	Sample, method and analysis

Nr	Authors	Year	Country	Technology	Approach	Stakeholders	Data collection methods	Topics	Challenges
59	Revenas et al.	2015	Sweden	Web-based and mobile app to support physical activity in individuals with rheumatoid arthritis	Participatory action research	Patients, HCPs, research experts, technical experts, advocacy group members	Workshop	Testing	Sample, method and analysis, social dynamic, topics
60	Stalberg et al.	2016	Sweden	"Inter-Active Communication Tool for Activities" [IACTA], is intended to facilitate young children's participation in healthcare situations	Participatory design	Patients	Heuristic evaluation	Requirements, design	Method and analysis
61	Terp	2018	Denmark	Smartphone app in the everyday management of living with schizophrenia:	Participatory design thinking and methods	Patients	Interview	Testing	Sample, method and analysis
62	Toefy et al.	2018	South Africa	A mobile phone, audio messaging intervention	Participative qualitative methods	Patients, relatives, communication expert	Focus group, not-protocolled meeting, think-aloud, heuristic evaluation, questionnaire	Background, requirements, designing, testing	Not reported
63	Tongpeth et al.	2018	Thailand	Avatar-based education application for patients with acute coronary syndrome	Participatory action research	Patients, HCPs, research experts	Not-protocolled meeting	Requirements, testing	Sample, method and analysis, social dynamic
64	Torres et al.	2019	India, Sri Lanka, Egypt, UAE, Mexico, UK, USA	Affordable Smart Wheelchair (ASW) a system that is accessible and affordable to users across the globe	Participatory design	Patients, HCPs	Interview	Background, requirements	Sample
65	Vacha and Kandusova	2018	Czech Republic	Smart Home Care system	Participatory design and multi-stakeholder collaboration	Patients, HCPs, citizens, relatives, technical experts, politicians	Workshop, heuristic evaluation	Testing	Sample
66	Van der Velden et al.	2016	Norway	Transition app: a mobile application that would support young patients during their transition to adult health care and the Transition Game: a game to support young patients in transition	Participatory Design: SHARM Framework (Situation-based action, Having a say, Adaptability, Respect, Mutual Learning)	Patients	Workshop, diary writing	Background, requirements, designing, testing	Method and analysis
67	Verbiest et al.	2019	Australia	Behavior change mHealth intervention for indigenous and other priority communities	Co-design	Citizens, research experts	Not-protocolled meeting, focus group	Background, requirements, testing, procedure	Sample
68	Voorend et al.	2019	The Netherlands	A personalized mobile application to help people with fertility problems to manage their condition and encourages a healthy lifestyle in order to improve the outcomes of fertility treatment	Co-design	Patients	Interviews, forum	Background, requirements	Not reported
69	Ward et al.	2015	UK	Warm Neighbourhoods service to help people live at home	Co-creation methodologies	Patients, HCPs, informal care givers, relatives, industry representatives	Interview, workshop	Requirements, testing	Not reported
70	Waterman	2018	Canada	A web-based app that helps youth in crisis to develop their own support plans, access community-based services, and encourage self-reflection as alternative methods of crisis-management	Collaborative design	Patients, HCPs	Interview, workshop	Background, requirements, testing, procedure	Social dynamic, topics
71	Wentzel et al.	2014	The Netherlands	A concept for a nurse information application	The CeHRes Road map / Participatory Design	Patients, HCPs, research experts, pharmasists	Focus groups, heuristic evaluation, observation	Problem, requirements	Sample, method and analysis, topics
72	Winterling et al.	2016	Sweden	Self-help web-based intervention targeting young cancer patients with sexual problems and fertility distress	Co-creative participatory process	Patients	Not-protocolled meeting	Background, requirements, designing, procedure	Sample, method and analysis, social dynamic
73	Wolpin et al.	2015	USA	Web-based cancer symptom and quality-of-life support intervention	Participatory design with a user-centred focus	Patients, informal care givers	Focus group, think-aloud	Background, designing, testing, procedure	Sample, method and analysis
74	Woods	2019	Australia	A Consumer Mobile Health App for Heart Failure: Care4myHeart	Co-Design	Patients, HCPs, informal care givers, relatives	Interview, workshop	Requirements, design, testing	Sample, method and analysis
75	Zachary et al.	2018	USA	Social networking app to support type II diabetes self-management in low-income minority communities	Participatory design	Patients, HCPs, technical experts	Forum	Background, requirements	Not reported

Abbreviations: USA = United States of America, UK = United Kingdom, HCPs = healthcare professionals, app = application

Table D2: Papers grouped in the involvement approach

Nr	Authors	Year	Country	Technology	Approach	Stakeholders	Data collection methods	Topics	Challenges
1	Amirabdollahian et al.	2014	UK, the Netherlands, Italy	A hand/wrist rehabilitation device for home-based therapy after stroke	User-centred design methodology	Patients, HCPs, informal care givers	Interview, focus group, think-aloud, heuristic	Testing	Not reported
2	Anglada-Martinez et al.	2017	Spain	Medplan, a platform for patients/caregivers and healthcare professionals	User-centered design	Patients, HCPs	Not-protocolled meeting	Requirements, design, testing	Feasibility, topics
3	Argent et al.	2018	Ireland	A prototype wearable exercise biofeedback system for orthopaedic rehabilitation	User-centred design	HCPs	Interview, questionnaire	Requirements	Feasibility, topics
4	Arvidsson et al.	2016	Sweden	Interactive assessment and communication tool for children with cancer	User-experience design	Patients, citizens, Parents, HCPs	Usability test, observation, interview	Background, testing	Sample
5	Baek et al.	2018	Korea	An mHealth Tool for Self-Management and Care Engagement of Cardiovascular Disease Patients	User research and user experience investigations	Patients, HCPs	Interview, focus group, heuristic evaluation	Requirements	Method and analysis, topics
6	Ben-Zeev et al.	2017	USA	FOCUS, a mobile system that delivers daily assessments and interventions designed to support self-management of their illness	A staged approach	Patients, HCPs	Focus group, think-aloud	Not reported	Sample, method and analysis, feasibility, topics
7	Bendixen et al.	2017	USA	App for people with brain and spinal cord anomalies	User-centred approach	Patients	Focus group	Not mentioned	Not reported
8	Bevan Jones et al.	2018	Whales	Web-based psychoeducational intervention for adolescent depression	Person-based or person centred approach	Patients, parents, informal care givers	Interview, focus group, workshop, not-protocolled meeting,	Requirements	Not reported
9	Birnie et al.	2019	Canada	Smartphone-based app for self-management of postoperative pain in children and adolescents	User-centred design	Patients, HCPs, research experts	Interview, focus group, delphi study, workshop	Background, requirements, designing, testing	Topics
10	Birnie et al.	2018	Canada	Achy Penguin, a parent-developed iOS app to help assess and manage acute pain in young children	User-centered design approach	Patients	Heuristic evaluation	Requirements, testing	Sample, social dynamics, topics
11	Blijleven et al.	2019	The Netherlands	A conceptual framework, SEWA, to address challenges of studying workarounds emerging from Electronic Health Record (EHR) system usage	Iterative process	HCPs, technical experts	Heuristic evaluation, questionnaire	Background, requirements, testing	Sample, method and analysis, feasibility
12	Borosund et al.	2018	Norway	Stress management app intervention for cancer survivors	User-centred design and service design methods	Patients, HCPs, technical experts	Interview, usability test	Background, requirements, testing	Sample, topics
13	Brox et al.	2017	Norway	Kinect exergame for seniors	User-centred design	Patients	Questionnaire, observation, interview, not-protocolled meeting, usability test	Requirements, designing, testing, procedure	Social dynamic
14	Buitenweg	2019	The Netherlands	An innovative, visual, and personalized QoL assessment app for people with severe mental health problems: the QoL-ME	User-centred design	Patients	Focus group	Problem, requirements, testing	Sample, feasibility
15	Cai et al.	2017	UK	Smartphone app system to improve self-management in young people with juvenile idiopathic arthritis	User-centred design	Patients, parents, HCPs	Focus group, interview	Requirements, designing, testing	Method and analysis
16	Calvillo-Arbizo et al.	2019	Spain	e-Health system for renal patients at home (AppNephro)	User-centred design approach	Patients, informal care givers, HCPs	Interview, questionnaire	Requirements, testing	Not reported
17	Connelly et al.	2016	USA	Ecological momentary assessment mobile app for a low-literacy, Mexican American population to collect disordered eating behaviors	User-centred, iterative design process	Patients, citizens	Focus group, interview	Background	Sample
18	Coons et al.	2019	USA	Medivate, a mobile app to collect medication list	Iterative, user-centred design process	Patients, HCPs	Interview, questionnaire, not-protocolled meeting	Background, testing	Not reported
19	Crehan et al.	2019	Malawi	NeoTree application: developing an integrated mHealth solution to improve quality of newborn care and survival	Iterative, user-centric development	HCPs, Student HCPs	Focus group, workshop, questionnaire	Testing	Not reported
20	Cristancho-Lacroix et al.	2019	France	A fully automated Web-based program for caregivers of PWAD (people with alzheimers disease)	User-centred design	Patients, HCPs, technical experts	Interview, workshop, heuristic evaluation	Requirements, testing	Sample, method and analysis, feasibility
21	Curtis et al.	2015	UK	Healthy eating app	User-centred design	HCPs, citizens	Not-protocolled meeting	Designing	
22	Danbjorg et al.	2018	Denmark	An Exercise App in the Care for People With Osteoarthritis	User-driven exploratory study	Patients	Focus group, heuristic evaluation, observation	Requirements, testing	Sample

Nr	Authors	Year	Country	Technology	Approach	Stakeholders	Data collection methods	Topics	Challenges
23	De La Vega et al.	2014	Spain	Painometer, a Smartphone app that helps users to assess pain intensity	Two iterative cycles	HCPs, citizens	Interview	Background, requirements, designing	Sample
24	Dimeff et al.	2018	USA	Avatar in EDs to perform a CAMS suicide risk assessment with suicidal ED patients, to teach behavioral skills for reducing imminent distress, and to generate hope by hearing personal stories from persons with lived experience	Iterative user-centred design	Patients, HCPs	Interview, usability test	Background, requirements, testing	Sample
25	Dirin et al.	2015	Finland	A Context-Aware Nurse Assistant (CANA)	User-centred design	HCPs	Interview, think-aloud, questionnaire	Requirements, designing, testing	Not reported
26	Dorrington	2016	UK	Assistive Switch Devices for electromyography standalone switch users who have complex disabilities	User-centred design	Patients	Interview	Background, designing, testing	Method and analysis, feasibility
27	Dowd et al.	2018	Canada	Self-regulatory app to effectively manage celiac disease	Participatory approach/user-centred design	Patients, HCPs	Focus group, questionnaire	Background, requirements, testing	Feasibility
28	Edmonds et al.	2019	USA	A decision support tool for expectant parents facing threatened periviable delivery	User-centred design	Patients, relatives	Not-protocolled meeting	Designing	Sample
29	Fernon et al.	2016	Australia	eTool for gout management	User-centred design	Patients	Focus group	Background, testing	Sample
30	Fledderus et al.	2015	Netherlands	Online relapse-prevention program based on acceptance and commitment therapy for chronic pain patients	User-centred design	Patients, technical experts	Focus group, interview, think-aloud	Requirements, testing	Sample
31	Fortuna et al.	2017	USA	Psychosocial intervention for smartphone delivery to middle-aged and older adults with serious mental illness	User-centred design	Patients, HCPs, technical experts	Not-protocolled meeting, usability test	Designing, testing	Sample, method and analysis, feasibility
32	Gabrielli et al.	2017	Italy	Mobile app for nutrition education (TreC-LifeStyle)and formative evaluation with families of overweight children	User-centred design	Parents, HCPs, research experts	Not-protocolled meeting, interview	Background, requirements, designing	Sample
33	Garzo et al.	2018	France	Gait training system for Parkinson’s disease	User-centred design	Patients, citizens, technical experts, consultants	Interview, observation, workshop, think-aloud	Background, requirements, designing, testing	Feasibility, topics
34	Geraghty et al.	2016	UK, United States, Spain, China and India	Internet-delivered intervention for emotional distress in primary care patients	Person-based approach	Patients	Interview, think-aloud	Background, testing	Sample
35	Ghysels et al.	2017	Belgium	A digital tool ‘H-OPP’ was developed to make the tool easier, more applicable and practical for use	Client-centred approach	Research experts	Not-protocolled meeting, questionnaire, observation	Requirements, designing, testing, procedure	Sample
36	Gill et al.	2019	Canada (British Columbia)	Mobile technology intervention to support postabortion care	User-centred design	Patients	Interview	Testing	Sample, feasibility
37	Giordanengo et al.	2018	Norway	Prototype for extracting relevant information and documenting information gaps from self-collected health data by patients with type 1 diabetes	User-centred design	Patients, HCPs	Workshop	Requirements, procedure	Sample
38	Gracey et al.	2018	USA	myEczema, a smartphone application for patient-reported outcomes in atopic dermatitis	User-centred design	Patients, HCPs, informal care givers, technical experts	Interview, focus group	Background, requirements	Not reported
39	Gray et al.	2016	Canada	Electronic Patient-Reported Outcomes Tool	User-centred design	Patients, HCPs, informal care givers, research experts, technical experts, content experts	Interview, focus group	Requirements, testing	Sample, method and analysis, social dynamics, feasibility, topics
40	Han et al.	2018	South Korea	DrugTEAM” (Drug Therapy Evaluation And Management), a Patient-oriented, Collaborative, Advanced, Renovated, and Excellent (P-CARE) service	Focus group discussions, interviews, observations	Patients, HCPs	Interview, focus group, questionnaire	Requirements, testing	Sample
41	Hanschke et al.	2017	UK	A social machine (echnology-enabled social systems) for the Heart Manual service	User-centred design	HCPs	Interview, workshop, think-aloud, brainstorm	Testing	Sample, method and analysis, feasibility
42	Hardy et al.	2018	UK	SlowMo, is an innovative blended digital therapy for people who fear harm from others	User-centred design	Patients, HCPs	Interview, observation	Background, requirements, procedure	Not reported
43	Harricharan et al.	2015	UK	myPace for weight management	Systematic, iterative stages of engagement	Patients, HCPs	Heuristic evaluation	Background, testing	Not reported

Nr	Authors	Year	Country	Technology	Approach	Stakeholders	Data collection methods	Topics	Challenges
44	Harte et al.	2017	Ireland	Mobile phone app in an integrated falls risk detection system for use by older adult users	Human-centred design	Patients, research experts, technical experts	Not-protocolled meeting, usability test, think-aloud, interview, questionnaire,	Background, testing	Sample, method and analysis
45	Haynes et al.	2016	USA	A mobile platform for care coordination for complex, chronic heart disease	User-centred design	Patients, HCPs, informal care givers	Interview, questionnaire	Background, testing	Not reported
46	Haynes et al.	2017	USA	A mobile platform for care coordination for complex, chronic heart Disease	User-centred design	Patients, HCPs	Think-aloud, heuristic evaluation	Requirements	Sample, social dynamic
47	Heynsbergh et al.	2019	Australia	Smartphone app for informal carers of people with cancer: processes and learnings	User-centred design / co-design process	Relatives, informal care givers	Interview, focus group, usability test	Background, testing	Sample
48	Holtz et al.	2017	USA	mHealth app: a tool for adolescents with type 1 diabetes and their parents	Patient-centred research methods	Patients, parents, informal care givers, HCPs, research experts, advocacy group members	Focus group	Background, testing	Not reported
49	Honary et al.	2018	UK	Web-based intervention for relatives of people experiencing psychosis or bipolar disorder	User-centred approach and heuristic evaluation	Relatives, informal care givers	Workshop, think-aloud, not-protocolled meeting	Background, requirements, designing, testing	Sample
50	Howarth et al.	2019	UK, United States, Spain, China and India	e-Health intervention for the workplace developed	Person-based approach	Citizens	Focus group, interview, think-aloud, workshop	Background, requirements, designing	Not reported
51	Hu et al.	2019	Canada	Mobile phone app for the self-management of pediatric concussion	User-centred design approach and agile development methods	Patients, HCPs	Interview, think-aloud	Testing	Sample
52	Huberty et al.	2016	USA	Text4baby is a free, mobile health information service for pregnant and post-partum women	User-centred and an iterative design process	Patients, HCPs	Interview	Background, testing	Sample
53	Jalil et al.	2019	Australia	Telehealth in-home monitoring device to manage type 2 diabetes mellitus from home	Clinical user-experience evaluation from a human computer interaction perspective	Patients	Interview, think-aloud	Background, testing	Not reported
54	Jibb et al.	2017	Canada	A mHealth Real-Time Pain Self-Management App for Adolescents With Cancer	User-centered design approach, iterative cycles of usability testing and interviews	Patients	Think-aloud, heuristic evaluation	Requirements, design, testing	Sample, method and analysis,
55	Jie et al.	2019	The Netherlands	"Stappy", a sensorfeedback system to facilitate walking in people after stroke	User-centred approach	Patients	Usability test	Testing	Sample
56	Joshi et al.	2019	USA	A diet app that would assist in management of patients with metabolic syndrome	Human centered approach	HCPs	Interview	Background	Not reported
57	Kip et al.	2019	The Netherlands	A VR application for treatment in forensic mental healthcare	CeHRes Roadmap	Patients, HCPs, technical experts, managers	Interview	Background, requirements, testing	Sample, feasibility, topic
58	Lau et al.	2018	Singapore	Healthy lifestyle mobile app for overweight pregnant women	User-centred approach	Patients	Interview	Background, requirements, testing	Sample, feasibility
59	Madrigal-Cadavid	2019	Colombia	Mobile app of drug information for people with visual impairment	User-centred design	Patients	Interview	Requirements	Not reported
60	Maher et al.	2015	USA	The BMT Roadmap, a portable, tablet-based platform	User-centred design	Patients, informal care givers	Workshop, questionnaire	Requirements, testing	Sample, social
61	Marien et al.	2019	Belgium	Web-based medication reconciliation application integrated in an eHealth network	User-centred study	HCPs	Interview, focus group, observation	Testing	Sample, feasibility
62	Martinez et al.	2016	Costa Rica	A software platform for the support of personalized cancer treatment	User-centred design	Patients, HCPs, technical experts, research experts	Not-protocolled meeting	Requirements, designing	Feasibility
63	Mateo et al.	2018	USA	Technology-assisted intervention designed to provide primary care-based 5As counseling within patient-centred medical homes	User-centred design	Patients, HCPs	Focus group, interview, think-aloud	Requirements, designing, testing	Sample
64	Mawson et al.	2014	UK, United States, Spain, China and India	Personalised self-managed rehabilitation system (PSMrS)	Hybrid of health and social sciences research methods and user-centred design methods and realist evaluation	Patients, relatives, informal care givers, HCPs	Focus group, think-aloud, workshop, interview	Background, requirements, testing	Not reported

Nr	Authors	Year	Country	Technology	Approach	Stakeholders	Data collection methods	Topics	Challenges
65	McGrath et al.	2017	USA	Concussion assessment mHealth app for certified Athletic Trainers	CeHRes Roadmap	Citizens	Focus group, delphi study	Requirements, designing, testing	Feasibility
66	Milward et al.	2016	UK	App to reduce harmful drinking in young adults	User-centred design	Patients	Focus group	Requirements, testing	Sample, topics
67	Mortsenson et al.	2019	Canada	Self-management app for people with spinal cord injury	User-centred design	Patients, relatives, HCPs	Focus group, interview, questionnaire	Requirements, designing, testing	Method and analysis, feasibility
68	Neubeck et al.	2015	Australia	e-Health tool for people with, or at high risk of, cardiovascular disease	Iterative process incorporating user-centred design	Patients	Focus group, usability test	Background, designing, testing	Sample
69	Nkoy et al.	2019	USA	Home monitoring system for children with medical complexity	User-centred design	Informal care givers	Focus group	Background, requirements	Sample, method and analysis
70	Olney et al.	2019	USA	Comprehensive mobile assessment of pressure (CMAP)system for pressure injury prevention for veterans with spinal cord injury	Experience based design	Patients	Focus group, interview	Requirements, testing	Sample
71	Oulton et al.	2018	UK	Informative game for preparing children for blood tests	User-experience design	Patients	Workshop, sketching	Requirements	Sample, social dynamics, feasibility, topics
72	Paay et al.	3028	Denmark	Interactive technologies supporting young adults in managing low self-esteem	User-centred study	Patients	Interview, focus group, workshop	Testing	Social dynamics, topics
73	Paulino et al.	2019	Portugal	An integrative system for Exergaming targeting the senior population	Human-centred design	HCPs	Interview, questionnaire	Testing	Method and analysis, topics
74	Pazart et al.	2017	France	A new medical device, the VEDIAS system, Device to Assist Manual Ventilation	User-centred design	HCPs	Heuristic evaluation	Background, testing	Method and analysis, topics
75	Prince et al.	2019	Canada	Web-based tool to support management of chemotherapy-related toxicities in cancer patients	User-centred participatory design methodology	Patients, informal care givers, HCPs	Interview, focus group, observation	Background, requirements, procedure	Sample, method and analysis
76	Rogers et al.	2019	USA	Composer, a visual analysis tool for orthopedic surgeons to compare changes in physical functions of a patient cohort following various spinal procedure	User-centred design	HCPs, Student HCPs, technical experts	Not-protocolled meeting	Requirements	Not reported
77	Rothangel et al.	2017	Netherlands, Germany	Telerehabilitation platform for patients with phantom limb pain	Iterative user-centred design	Patients, HCPs, Student HCPs, technical experts	Questionnaire, interview, not-protocolled meeting, usability test	Requirements, testing	Sample, topics
78	Runaas et al.	2017	USA	"BMT Roadmap" is a web-based application that integrates patient-specific information	User-centred design / patient-centred design	Informal care givers	Interview, observation	Background, requirements, testing	Sample
79	Schnall et al.	2016	USA	A mHealth app for persons living with HIV	User-centred design	Patients, technical experts	Heuristic evaluation	Background, requirements, designing, testing	Not reported
80	Schneider et al.	2016	USA	Mobile app to aid disease self-management	Patient-centred design/user-centred design	Patients	Interview	Testing	Not reported
81	Schwartz et al.	2019	USA	A Tailored mHealth Intervention for Adolescent and Young Adult Survivors of Childhood Cancer	Agile development	Patients, HCPs, citizens, technical experts	Focus group, not-protocolled meeting	Requirements	Sample, method and analysis
82	Sedlmayr et al.	2019	Germany	Mobile interface concept to support the management of medication information	Human-centred design process for interactive systems	Patients, research experts, technical experts	Interview, observation, usability test, think-aloud, interview	Background, requirements, testing	Topics
83	Sewitch et al.	2019	Canada	A Smartphone App That Supports Colonoscopy Preparation	User-centred approach	Patients	Focus group, observation	Testing	Sample
84	Schellmer et al.	2017	USA	Teen Pocket PATH, a mobile health application to improve medication adherence in adolescent solid organ recipients	User-centred design	Patients, informal care givers	Heuristic evaluation	Testing	Sample, social dynamic, feasibility
85	Sivan et al.	2016	UK	Home-based Computer Assisted Arm Rehabilitation (hCAAR), a home-based robotic device	User-centred design	Patients, HCPs	Interview	Testing	Sample, method and analysis, topic
86	Smaradottir et al.	2015	Norway	Information system for a Norwegian telemedicine service	User-centred design	Patients, HCPs, technical experts, advocacy group members	Interview, think-aloud, workshop	Requirements, testing	Not reported

Nr	Authors	Year	Country	Technology	Approach	Stakeholders	Data collection methods	Topics	Challenges
87	Sobrinho et al.	2018	Brazil	Mobile application to assist the self-monitoring of the chronic kidney disease	User-centred design	Patients, HCPs	Interview	Requirements, designing, testing	Not reported
88	Srinivas et al.	2017	USA/Singapor e	Consumer health IT application for geriatric heart failure selfcare	User-centred design	Patients, relatives, informal care givers	Interview, think-aloud	Backrgound, testing	Not reported
89	Tamblyn	2017	Canada	Computer-assisted tool with automated electronic integration of population-based community drug data: the RightRx project	User-centred design and agile development processes	HCPs	Workshop	Requirements	Not reported
90	Thirumalai et al.	2018	USA	Telerehabilitation mHealth app	User (participant)-centred design	Patients	Focus group, think-aloud	Requirements, testing	Sample, topics
91	Timmerman et al.	2016	Netherlands	ICT-supported cancer rehabilitation application for resected lung cancer survivors	User-centred approach	Patients, HCPs	Interview, focus group, not-protocolled meeting,	Requirements, testing	Sample, topics
92	Tonkin et al.	2017	Australia	Smartphone app to reduce sugar-sweetened beverage consumption among young adults in Australian remote indigenous communities	Consultative user-centred approach	Citizens	Interview, think-aloud, questionaire, workshop	Background, requirements, designing	Sample, method and analysis, feasibility
93	Wachtler et al.	2018	Australia	Mobile clinical prediction tool to estimate future depression severity and guide treatment in primary care	User-centred design	Patients	Interview, focus group	Requirements, testing	Sample
94	Wilcox et al.	2016	USA	myNYP Inpatient a custom personal health record application	User-centred approach	Patients, HCPs	Interview	Requirements, testing	Sample
95	Willard et al.	2018	Netherlands	Online community care platform for frail older adults in the Netherlands	User-centred design	Patients	Interview, observation	Background, requirements, testing	Not reported
96	Williamson et al.	2014	USA	A Mobile/Web App for Long Distance Caregivers of Older Adults	User-centred design	Patients	Interview, think-aloud	Testing	Sample, social dynamic, topics
97	Wozney et al.	2015	Canada	Breathe - An Internet-based, CBT treatment program for anxious adolescents with mild to moderate distress and impairment	Lean user experience design	Patients, HCPs	Interview, heuristic evaluation	Background, requirements, designing	Sample
98	Yoo et al.	2015	South Korea	The Smart Bedside Station (SBS) is a screen that is installed on the bedside for the personal use and provides a variety of convenient services for the patients	User experience design	Patients, HCPs, research experts	Interview, workshop	Background, requirements, testing	Sample
**Abbreviations: USA = United States of America, UK = United Kingdom, HCPs = healthcare professionals, app = application**									

Table D3: Papers grouped in the consultation approach

Nr	Authors	Year	Country	Technology	Approach	Stakeholders	Data collection methods	Topics	Challenges
1	Abrams et al.	2018	USA	he Bridge Mobile Application (App) for Burn Patients	Qualitative inquiry: three focus groups	Patients, HCPs	Focus group	Background, requirements	Sample
2	Acharya and Sarraf	2017	USA	A novel Android application (Pro-Care) that provides a real-time, multi-way communication between patients and providers	Practical evaluation	Patients, relatives	Not-protocolled meeting	Requirements, testing	Not reported
3	Ahmadi et al.	2015	Iran	A Software for Teaching Health Related Topics to Deaf Students	Descriptive study using interviews	Hygiene instructors	Interview	Problem, requirements, procedure	Feasibility, topics
4	Ahmed et al.	2015	Malawi	The Knowledge for Health (K4Health) Project	A needs assessment	Patients, HCPs	Interview, focus group	Testing	Method and analysis, social dynamic, feasibility, topics
5	Aliabadi et al.	2016	USA	An HIV Prevention Smartphone Application for High-Risk MSM	Focus group sessions	Patients	Focus group	Background, requirements	Sample, method and analysis, feasibility, topic
6	Alnasser et al.	2015	Saudi Arabia	Weight loss application	Focus group	Patients	Focus group	Background	Not reported

Nr	Authors	Year	Country	Technology	Approach	Stakeholders	Data collection methods	Topics	Challenges
7	Alnosayan et al.	2017	USA	mHealth system that could effectively support heart failure	Design science research	Patients, HCPs, technical experts	Interview, observation, focus group, questionnaire	Requirements, testing	Sample, method and analysis
8	Angula and Dlodlo	2016	Nambia	A Mobile Application for Health Information Dissemination	Qualitative approach	HCPs, citizens	Interview, questionnaire	Background, requirements	Not reported
9	Anshari et al.	2015	Brunei	Clinic 2.0 - facilitate interactions between healthcare providers and customers	Mixed methods, qualitative	Patients, HCPs	Interview, questionnaire	Background, requirements, designing, procedure	Not reported
10	Armin et al.	2017	USA	The See Me Smoke-Free™ (SMSF) mobile health application	Focus groups	Patients	Focus group	Requirements	Sample, topics
11	Athilingam et al.	2018	USA	An Interactive Mobile Health Application to Improve Self-care in Heart Failure (HeartMapp)	Intervention Mapping Approach / Needs assessment	Patients, HCPs	Interviews	Background, requirements	Not reported
12	Baier et al.	2015	USA	Web-based customizable report platform	Multiphased approach	Patients, relatives, HCPs	Interview, focus group	Background, requirements, testing	Sample
13	Biedinger-Friedman et al.	2016	USA	Smartphone app designed to improve health behaviors among participants in the special supplemental nutrition program for women, infants, and children	Focus group	Patients	Focus group	Background	Sample, method and analysis
14	Bishop et al.	2016	UK	A new evidence-based website about acupuncture for back pain	Qualitative "think aloud" audio-recorded interviews	Patients	Think-aloud	Background, requirements, testing	Sample, topics
15	Bobin et al.	2018	France	A self-contained smart cup that can be used to perform exercises that are similar to everyday tasks such as drinking	Semi-structured interviews	Patients, HCPs, technical experts	Interview	Background, requirements, testing, procedure	Method and analysis
16	Casida et al.	2018	USA	A mobile phone application (VAD Care App) to organize and simplify the left-ventricular assist device self-management process	Interviews	Patients, HCPs	Interview	Background, requirements, testing	Sample, method and analysis
17	Compagna and Kohlbacher	2015	Germany	Service robots in care facilities for older people	Scenario-based design (SBD) method - interviews and focus groups	Patients, HCPs, managers	Interview, focus group	Background, requirements, procedure	Sample, method and analysis, social dynamic
18	Deighan et al.	2017	UK	Digital version of the heart manual: cardiac rehabilitation programme	Mixed method design	Patients, HCPs, research experts, technical experts	Interview, not-protocolled meeting, questionnaire	Testing	Not reported
19	Den Bakker et al.	2019	Netherlands	Electronic health program to empower patients in returning to normal activities after general surgical and gynecological procedures	Intervention mapping framework	Patients	Focus group, questionnaire	Requirements	Sample
20	Doarn et al.	2019	USA	iPad app for smoking cessation counseling and shared decision making in primary care	Conceptual framework grounded in system development methodology and behavioral theories	Patients, HCPs, research experts, advocacy group members	Focus group, interview	Requirements	Not reported
21	Domanska et al.	2017	UK	Certolizumab pegol electromechanical selfinjection device	Human factors studies	Patients, citizens, infromal care givers, HCPs	Interview	Designing, testing	Method and analysis
22	Dworkin et al.	2018	USA	Realistic talking human embodied agent mobile phone intervention to promote HIV medication adherence and retention in care in young HIV-positive African American men who have sex with men	Focus group	Citizens	Focus group	Requirements	Sample
23	Edwards et al.	2018	UK	Cigbreak smoking cessation mobile phone game	Agile development	Patients	Focus group	Background, requirements, testing	Method and analysis, social dynamic, feasbility
24	Ehrler et al.	2019	Switzerland	Mobile phone app for bedside nursing care	Software development life cycle model	HCPs	Focus group, usability test	Background, requirements, testing	Sample
25	Escalada-Hern et al.	2019	Spain	Prototype of augmented reality applied to medical devices	Rapid application development methodology	HCPs	Focus group, questionnaire, think-aloud	Requirements, testing	Not reported
26	Fager et al.	2017	USA	Sensor access system for environmental control for people with disabilities	Focus group	Patients, HCPs	Focus group	Testing	Not reported
27	Gilbert et al.	2016	Canada	Internet-based testing program for sexually transmitted and blood-borne infections	Holistic approach developing ehealth technologies	Patients, citizens	Focus group, interview, questionnaire	Requirements, testing	Feasibility

Nr	Authors	Year	Country	Technology	Approach	Stakeholders	Data collection methods	Topics	Challenges
28	Ginsburg et al.	2016	Ghana	Mpneumonia, an innovative mHealth application for diagnosing and treating childhood pneumonia	Field usability testing	HCPs	Think-aloud	Testing	Not reported
29	Gogovor et al.	2016	Canada	Internet-based chronic pain self-management program	Focus group	Patients, HCPs	Focus group	Background, requirements	Sample
30	Goodwin et al.	2016	Ireland	Mental health smartphone app	Qualitative descriptive design	Patients	Interview	Requirements	Sample, social dynamic
31	Gordon et al.	2017	USA	mHealth app to increase tobacco cessation medication adherence	Focus groups (think aloud)	Patients	Focus group	Designing, testing	Sample
32	Grasaas et al.	2019	Norway	Self-management app for adolescents with persistent pain in Norway	Usability test in laboratory setting and field usability test	Patients	Usability test, think-aloud, interview	Testing	Sample, method and analysis, feasibility
33	Gray et al.	2019	UK	Electronic personal assessment questionnaire for menstrual, pelvic pain and gynaecological hormonal disorders	Interviews and panel review	Patients	Interview,	Background, requirements, testing	Sample
34	Grindell et al.	2018	UK	Online programme to promote walking	Pragmatic, iterative process of qualitative data collection and analysis with distinct phases and differing user groups	Patients, teachers	Focus group, interview	Testing	Sample, method and analysis
35	Hardinge et al.	2015	UK	An internet-linked tablet computer based mobile health (mHealth) system in improving quality of life in patients with moderate to severe COPD	Participant informant group	Patients, HCPs	Focus group, workshop	Background, requirements, designing, testing	Not reported
36	Hasin et al.	2014	USA	HealthCall for the smartphone: intervention in HIV alcohol dependent patients	Focus group and multidisciplinary team	Patients	Interview	Background, requirements	Sample, topic
37	Hill et al.	2016	Australia	eSALT, an asynchronous telerehabilitation platform for speech-language pathology	Mixed methods	Patients, HCPs	Heuristic evaluation	Testing	Sample
38	Hohenstein et al.	2017	USA	Optical reader system to support point-of-care rapid diagnostic testing	Iterative design methodology	Citizens	Interview, think-aloud	Testing	Sample, social dynamic, feasibility, topics
39	Houze de l'Aulnoit et al.	2018	France	A Smart Mobile Data Module for Fetal Monitoring in E-Healthcare	Interviews	Patients, HCPs	Interview	Background, requirements	Not reported
40	Hsieh et al.	2018	USA	A Fall Risk mHealth App for Older Adults	Iterative design-evaluation process of semistructured interviews	Citizens	Interview	Requirements, designing, testing	Sample, method
41	Joe et al.	2017	USA	IT-based wellness tools for older adults	6-8-5 design method	Patients	Focus group	Requirements, designing	Sample, method and analysis, topics
42	Kabeza et al.	2019	Rwanda	Diabetes self-management smartphone application (Kir'App)	Qualitative study design	Patients	Interview	Requirements	Sample, method and analysis, topics
43	Kim et al.	2015	USA	Tablet Device App for Parkinson's Disease Patients' Continuous Self-Monitoring and Management	Interviews	Patients, HCPs	Interview	Requirements	Not reported
44	Knight-Agarwal et al.	2015	Australia	Eating4two mobile phone app to monitor gestational weight gain	Qualitative investigation supported by an evidence-based approach	Patients, HCPs, research experts	Focus group, interview, not-protocolled meeting	Background, requirements, testing	Sample, method and analysis
45	Kohlstadt et al.	2015	USA	Online, peer leader component to an existing in-person preventive nutrition intervention called NutriBee	Focus group	Patients, parents, HCPs	Focus group	Requirements	Not reported
46	Krishnamurti et al.	2017	USA	MyHealthyPregnancy App: a behavioral decision research-based tool for assessing and communicating pregnancy risk	Interview	Patients, HCPs, research experts	Interview	Background, testing	Sample
47	Kristiansen et al.	2017	Denmark	An educational website for patients with heart failure	A two-step qualitative study	Patients	Focus group, workshop, diary writing	Requirements, designing	Sample
48	Lui et al.	2017	China	"Information Assistant": A Smartphone Application to Meet the Personalized Information Needs of Women with Breast Cancer	Interviews, experts group discussion, and e-Delphi studies	Patients	Interview, focus group, delphi study	Requirements	Sample, method and analysis

Nr	Authors	Year	Country	Technology	Approach	Stakeholders	Data collection methods	Topics	Challenges
49	Lwin et al.	2016	Singapore	Social media mhealth solution to address the needs of dengue prevention and management in Sri Lanka	Interview	Inspectors	Interview	Background, requirements	Not reported
50	Marsac et al.	2015	USA and Australia	Innovative, preventive web-based game for children exposed to acute trauma	Interview	Patients, parents	Interview	Testing	Sample, method and analysis, topics
51	Martinez et al.	2018	USA	Patient-facing diabetes dashboard embedded in a patient web portal	Interview and meeting	Patients, HCPs, research experts	Not-protocolled meeting, questionnaire	Requirements, testing	Sample, method and analysis, social dynamic
52	Materia	2018	USA	Smartphone intervention for women with overweight and obesity	Interview and focus group	Patients	Focus group, interview	Background, requirements	Sample, method and analysis
53	Mattie et al.	2019	Canada	Kneeling wheelchair with "on the fly" adjustable seating functions	Open ended questions in questionaire	Patients	Questionnaire	Testing	Sample, method and analysis, feasibility
54	McClelland and Fitzgerald	2018	UK	A mobile app for mental health service users and clinicians	Qualitative design using four focus groups	Patients, HCPs	Focus group	Requirements, designing	Sample, feasibility
55	McDonald et al.	2017	UK	A Web-Based Diary Tool for Self-Monitoring Symptoms in Glaucoma	Prospective mixed-method feasibility study	Patients	Diary writing	Testing	Sample
56	Middelweerd	2018	Netherlands	App-based Intervention to promote physical activity among young adults	Pilot testing	Citizens	Usability test	Testing	Not reported
57	Milward et al.	2017	UK	The app “BRANCH” targets harmful drinking in young adults (18-30 year olds)	Usability Testing / Focus groups	Citizens	Focus group	Testing	Sample, social dynamic
58	Mohamad Marzuki et al.	2019	Malaysia	Usable mobile app for community education on colorectal cancer	Nominal group technique	HCPs	Nominal group technique	Requirements	Not reported
59	Morrison et al.	2019	Malaysia	A mobile application is being developed to help reduce stress and uncertainty for families who have a child newly diagnosed with cancer	A 2-phase qualitative study using the design thinking approach of product ethnography	Patients	Interview	Background, requirements	Sample, topics
60	Nguyen et al.	2018	Australia	mHealth app patient testing and review of educational materials designed for self-management of gout patients	Interview and focus group	Patients	Focus group, interview	Testing	Sample, feasibility, topics
61	Ning et al.	2019	China	Parenting app to prevent unintentional injury in newborn babies and toddlers	2-step sequential mixed-method study	Relatives, teachers	Focus group	Background, requirements	Sample, social dynamic
62	Nitsch et al.	2016	Austria	Guided online and mobile self-help program for individuals with eating disorders	Iterative usability study design approach	Patients	Interview, think-aloud	Testing	Method and analysis, social dynamic, topics
63	O'Reilly et al.	2018	Australia	Smartphone app design for diabetes prevention in women with previous gestational diabetes	Focus group	Patients	Focus group	Testing	Social dynamic
64	Ogden et al.	2019	UK	App (Ladle)for weight loss and behaviour change	Needs assessment, experts reviews, focus groups, ongoing feedback	Patients, HCPs, research experts, advocacy group members, consultants	Focus group, interview, not-protocolled meeting	Requirements, testing	Not reported
65	Pearon et al.	2016	UK	Web-based version of an exercise-based rehabilitation program for people with chronic knee and hip pain	Focus groups and semistructured think aloud interviews	Patients	Focus group, interview, think-aloud	Testing	Sample, feasibility
66	Piau et al.	2015	France	Smart insole to promote healthy aging for frail elderly individuals	Focus group	Patients, HCPs	Focus group	Requirements, testing	Not reported
67	Pilco et al.	2019	Equador	The ePHoRT platform allows hip arthroplasty patients recovering from fracture or joint replacement to carry out part of the rehabilitation treatment at home	Usability evaluation	Technical experts	Heuristic evaluation	Testing	Not reported
68	Possemato et al.	2017	USA	PTSD Coach app - a self-management mobile app	Qualitative interviews	HCPs	Interview	Testing	Sample
69	Pramana et al.	2014	USA	The SmartCAT: An m-Health Platform for Ecological Momentary Intervention in Child Anxiety Treatment	Feasibility study	Patients, HCPs, technical experts	Heuristic evaluation	Testing	Sample
70	Prapkree et al.	2019	USA	Snackability smartphone application to identify healthy and unhealthy snacks	Analysis, design, development, implementation, and evaluation model	Citizens	Focus group	Background, testing	Sample

Nr	Authors	Year	Country	Technology	Approach	Stakeholders	Data collection methods	Topics	Challenges
71	Prokhorov	2017	USA	Mobile phone text messaging program for tobacco risk communication among college students	Focus group, mixed methods	Citizens	Focus group	Testing	Sample, method and analysis
72	Puijk-Hekman et al.	2017	Netherlands	Self-management web-based support program for patients with cardiovascular diseases	Intervention mapping framework	HCPs	Not-protocolled meeting	Requirements, designing, procedure	Sample, social dynamic
73	Reese et al.	2017	USA	Text messages to promote physical activity among African American women attending college	Focus groups/nominal group technique	Patients	Focus group	Requirements, testing	Sample, social dynamic
74	Ribigan et al.	2018	Romania and Belgium	Follow.Me system	Multi-structured approach / user needs and requirements analysis	Patients, HCPs	Interview, questionnaire	Testing	Not reported
75	Rivera et al.	2018	Canada	Mobile app for weight and health management in adolescents with complex health needs	Descriptive qualitative research design	Citizens, relatives, informal care givers, HCPs	Focus group	Background, requirements, procedure	Sample
76	Rohde et al.	2019	Germany	Mobile app intervention to improve eating habits of adolescents and young adults	Behavior change wheel	Patients, citizens, HCPs, research experts, technical experts, marketing experts	Interview	Background, requirements	Sample, feasibility, topics
77	Salgado et al.	2018	USA	Interactive Mobile Health and Rehabilitation (iMHere), Medication Management Smartphone App	The Delphi method	HCPs, informal care givers	Delphi	Design	Sample, method and analysis, topic
78	Sauer et al.	2018	USA, France, Germany, Brazil and South Korea	New pen device for injection of recombinant human growth hormone	Interview	HCPs	Interview	Testing	Sample
79	Shepherd et al.	2015	New Zealand	Computerized gamified depression therapy program for indigenous Māori adolescents	Focus groups using kaupapa Māori methodology	Citizens	Focus group	Testing	Sample, topics
80	Smith-Turchyn et al.	2017	Canada	TAPESTRY-CM healthy lifestyle app, an online self-management application for older adults with diabetes and hypertension	Cognitive interviewing techniques	Patients	Interview, workshop	Testing	Sample, method
81	Soomro et al.	2019	Australia	Injury Surveillance App for Cricket: TeamDoc is the first complementary, standalone mobile app that records cricket injuries through a smartphone	Survey with open answers	Citizens	Questionnaire	Designing, testing	Not reported
82	Stinson et al.	2014	Canada	Web- and mobile-based self-management program for youth with chronic pain	Descriptive exploratory qualitative design	Patients, HCPs	Focus group, interview	Background, requirements, testing	Sample, method and analysis
83	Teh et al.	2018	Australia	Handheld health information technology tool to support the timely update of bedside visual cues to prevent falls in hospitals	Focus group	HCPs	Focus group	Testing	Sample, method and analysis, social dynamic, feasibility
84	Thilo et al.	2019	Switzerland	Wearable fall detection device	Real field testing approach underpinned by the theoretical framework “medical device technology development process"	Patients	Focus group, not-protocolled meeting	Testing, procedure	Sample, feasibility
85	Thomas et al.	2019	UK	Digital group fatigue management program	Interviews and consultation groups	Patients, HCPs, research experts, technical experts	Interview, not-protocolled meeting	Requirements, testing	Sample
86	Threatt et al.	2017	USA	Assistive robotic table (ART)for stroke patients	Iterative design and evaluation study using both qualitative and quantitative components in a mixed-methods research design	HCPs, research experts, technical experts	Delphi study, focus group, think-aloud, usability test	Requirements, designing	Sample, method and analysis
87	Timmons et al.	2017	USA	SmartphSmartphone app to support adolescents' use of long-acting reversible contraception	Interview	Patients	Interview	Testing	Sample, method and analysis, social dynamic
88	Tonheim and Babic	2019	Norway	A mobile application for patient self-management within the field of Multiple Sclerosis (MS)	Semi-structured interview	HCPs	Interview	Designing, testing	Not reported
89	Vanoh et al.	2018	Malaysia	An education tool that is web-based, called WESIHAT 2.0, had been created to educate elderly people about precautionary strategies against mild cognitive impairment	Survey with open answers	HCPs, technical experts	Questionnaire	Testing	Not reported
90	Villegas et al.	2014	Chile	Internet-based STI–HIV prevention intervention for young Chilean women	Panels and questionnaire	Citizens, research experts, technical experts	Not-protocolled meeting, questionnaire	Requirements, design	Sample, method and analysis

Nr	Authors	Year	Country	Technology	Approach	Stakeholders	Data collection methods	Topics	Challenges
91	Vorrink	2016	Netherlands	Mobile phone app to stimulate daily physical activity in patients with chronic obstructive pulmonary disease	Group consultation sessions	Patients, citizens	Focus group	Testing	Sample
92	Walker et al.	2017	Australia	Colorectal cancer RISk Prediction tool ('CRISP') for use in primary care.	Simulated consultations / interviews	HCPs	Interview	Designing, testing	Sample, method and analysis, social dynamic
93	Wang et al.	2016	China	''Care Assistant'': A smartphone application to support caregivers of children with acute lymphoblastic leukaemia	Key informant interviews and focus group studies	Informal care givers	Interview	Background, requirements	Sample, feasibility
94	Ward et al.	2016	Canada	Smartphone app as a patient education tool for the management of thalassemia and iron overload syndromes	Delphi method	Patients	Delphi method	Requirements	Not reported
95	Ward et al.	2017	UK	FallCheck is a web app that supports identification of home-hazards and directs users towards self-management strategies to reduce risk of falling	Qualitative survey	HCPs	Questionnaire	Requirements, design	Sample, topic
96	Warren et al.	2018	New Zealand	Smartphone-based app for behavior change for youth drivers	Semistructured phone interviews or less formal discussion-based sessions	Patients, parents, advocacy group members	Interview	Background, requirements	Not reported
97	Webster et al.	2016	UK	Intervention to increase condom use in heterosexual men (the MenSS website)	The Behaviour Change Wheel	Patients, HCPs	Interview, workshop	Requirements	Sample, method and analysis, topics
98	Weichtelt et al.	2019	USA	Application designed to assist clinicians in returning injured farmworkers to light-duty job assignments	Interview and focus group	Citizens	Focus group, interview	Background, requirements, testing	Sample, method and analysis
99	Werner-Seidler et al.	2019	Australia	The Sleep Ninja is a fully-automated app that delivers CBT-I to young people cognitive behavioral therapy for insomnia	This qualitative study involved 3 focus groups	Patients	Focus group	Background, requirements, design	Sample, method and analysis, topics
100	Whiteley et al.	2019	USA	Gaming app to increase adherence to pre-exposure prophylaxis	Interviews, iterative and collaborative procedures	Patients	Interview	Testing	Sample, feasibility
101	Wilson et al.	2019	USA	Multi-Component Mobile-enhanced Treatment for Smoking Cessation	Qualitative methodology	Patients	Interview, think aloud	Requirements, design, testing	Sample, method and analysis
102	Zafeiridi et al.	2018	Italy/Spain/France/UK	Web-based platform for people with memory problems and their caregivers (CAREGIVERSPRO-MMD)	Mixed method design	Patients, relatives, informal care givers, HCPs	Questionnaire	Testing	Feasibility, topics
103	Zieve et al.	2017	USA	Personalized feedback for multiple health risk behaviors and reinforcement for health promoting behaviors from an electronic health screening tool for primary care setting	Interview	Patients	Interview	Testing	Sample, method and analysis
104	Zuidema et al.	2015	Netherlands	Online tailored self-management program for patients with rheumatoid arthritis	Intervention mapping framework	Patients, HCPs, research experts	Focus group, not-protocolled meeting	Requirements, testing	Sample

Abbreviations: USA = United States of America, UK = United Kingdom, HCPs = healthcare professionals, app = application
