# Supplementary material for: An Overview of Stakeholders, Methods, Topics, and Challenges in Participatory Approaches Used in the Development of Medical Devices: A Scoping Review
Source: Int J Health Policy Manag. 2022 Nov 5;12:6839. doi: 10.34172/ijhpm.2022.6839 (PMC10125077; doi:10.34172/ijhpm.2022.6839)
Supplement: Supplementary file 6 — Literature List of Included Papers. [file ijhpm-12-6839-s006.pdf]

Article title: An Overview of Stakeholders, Methods, Topics, and Challenges in Participatory Approaches Used in the Development of Medical Devices: A Scoping Review

Journal name: International Journal of Health Policy and Management (IJHPM)

Authors' information: Kas Woudstra[1]*, Rob Reuzel[2], Maroeska Rovers[2], Marcia Tummers[2]

[1]Department of Health Evidence and Operation Rooms, Radboud University Medical Center, Nijmegen, The Netherlands.

[2]Department of Health Evidence, Radboud University Medical Center, Nijmegen, The Netherlands.

(*Corresponding author: Kas.Woudstra@radboudumc.nl)

Supplementary file 6. Literature List of Included Papers

1. Abrams TE, Lloyd AA, Elzey LE, Hickerson WL. The Bridge: A mobile application for burn patients. *Burn J Int Soc Burn Inj*. 2019;45(3):699-704. doi:10.1016/j.burns.2018.09.028.
2. Abrines Jaume N, Abbiss M, Wray J, Ashworth J, Brown KL, Cairns J. CHILDSPLA: a collaboration between children and researchers to design and animate health states. *Child Care Health Dev*. 2015;41(6):1140-1151. doi:10.1111/cch.12280.
3. Acharya S, Sarraf R. A novel preventative solution for effective asthma management: a practical evaluation. *Netw Model Anal Heal Informatics Bioinforma*. 2017;6(1). doi:10.1007/s13721-017-0156-7.
4. Ahmadi M, Abbasi M, Bahaadinbeigy K. Design and implementation of a software for teaching health related topics to deaf students: the first experience in iran. *Acta Inform medica AIM J Soc Med Informatics Bosnia Herzegovina Cas Drus za Med Inform BiH*. 2015;23(2):76-80. doi:10.5455/aim.2015.23.76-80.
5. Ahmed N, Limaye RJ, Harlan S V. A multilevel approach to knowledge sharing: Improving health services for families and children. *Ann Anthropol Pract*. 2015;39(2):192-204. doi:10.1111/napa.12079.
6. Aliabadi N, Carballo-Dieguez A, Bakken S, et al. Using the Information-Motivation-Behavioral Skills Model to Guide the Development of an HIV Prevention Smartphone Application for High-Risk MSM. *AIDS Educ Prev Off Publ Int Soc AIDS Educ*. 2015;27(6):522-537. doi:10.1521/aeap.2015.27.6.522.
7. Allin S, Shepherd J, Tomasone J, et al. Participatory Design of an Online Self-Management Tool for Users With Spinal Cord Injury: Qualitative Study. *JMIR Rehabil Assist Technol*. 2018;5(1):e6. doi:10.2196/rehab.8158.

8. Alnasser AA, Alkhalifa AS, Sathiaseelan A, Marais D. What Overweight Women Want from a Weight Loss App: A Qualitative Study on Arabic Women. *JMIR mHealth uHealth*. 2015;3(2):1-7. doi:10.2196/mhealth.4409.
9. Alnosayan N, Chatterjee S, Alluhaidan A, Lee E, Houston Feenstra L. Design and Usability of a Heart Failure mHealth System: A Pilot Study. *JMIR Hum Factors*. 2017;4(1):1-13. doi:10.2196/humanfactors.6481.
10. Amirabdollahian F, Ates S, Basteris A, et al. Design, development and deployment of a hand/wrist exoskeleton for home-based rehabilitation after stroke - SCRIPT project. *Robotica*. 2014;32(8):1331-1346. doi:10.1017/S0263574714002288.
11. Anglada-Martínez H, Martin-Conde M, Rovira-Illamola M, et al. An Interactive Mobile Phone-Website Platform to Facilitate Real-Time Management of Medication in Chronically ill Patients. *J Med Syst*. 2017;41(8):122. doi:10.1007/s10916-017-0767-7.
12. Angula N, Dlodlo N. A Mobile Application for Health Information Dissemination: a Namibian Context. *Int Conf Adv Comput Commun Eng*. 2016:461-466.
13. Anshari M, Almunawar MN. Designing role of online health educators in healthcare services. *J evidence-informed Soc Work*. 2015;12(2):220-236. doi:10.1080/15433714.2013.815595.
14. Argent R, Slevin P, Bevilacqua A, Neligan M, Daly A, Caulfield B. Clinician perceptions of a prototype wearable exercise biofeedback system for orthopaedic rehabilitation: a qualitative exploration. *BMJ Open*. 2018;8(10):e026326. doi:10.1136/bmjopen-2018-026326.
15. Armin J, Johnson T, Hingle M, Giacobbi PJ, Gordon JS. Development of a Multi-Behavioral mHealth App for Women Smokers. *J Health Commun*. 2017;22(2):153-162. doi:10.1080/10810730.2016.1256454.
16. Arvidsson S, Gilljam BM, Nygren J, Ruland CM, Nordby-Bøe T, Svedberg P. Redesign and validation of sisom, an interactive assessment and communication tool for children with cancer. *JMIR mHealth uHealth*. 2016;4(2):1-16. doi:10.2196/mhealth.5715.
17. Athilingam P, Clochesy JM, Labrador MA. Intervention Mapping Approach in the Design of an Interactive Mobile Health Application to Improve Self-care in Heart Failure. *CIN - Comput Informatics Nurs*. 2018;36(2):90-97. doi:10.1097/CIN.0000000000000383.
18. Baek H, Suh J-W, Kang S-H, et al. Enhancing User Experience Through User Study: Design of an mHealth Tool for Self-Management and Care Engagement of Cardiovascular Disease Patients. *JMIR cardio*. 2018;2(1):e3. doi:10.2196/cardio.9000.
19. Baier RR, Cooper E, Wysocki A, Gravenstein S, Clark M. Using Qualitative Methods to Create a Home Health Web Application User Interface for Patients with Low Computer Proficiency. *eGEMs (Generating Evid Methods to Improv patient outcomes)*. 2015;3(2):1-14. doi:10.13063/2327-9214.1166.

20. Baldereschi M, Di Carlo A, Piccardi B, Inzitari D. The Italian stroke-app: ICTUS3R. *Neurol Sci Off J Ital Neurol Soc Ital Soc Clin Neurophysiol*. 2016;37(6):991-994. doi:10.1007/s10072-016-2506-0.
21. Baskerville NB, Struik LL, Dash D. Crush the crave: development and formative evaluation of a smartphone app for smoking cessation. *J Med Internet Res*. 2018;6(3):1-14. doi:10.2196/mhealth.9011.
22. Ben-Zeev D, Kaiser SM, Brenner CJ, Begale M, Duffecy J, Mohr DC. Development and usability testing of FOCUS: a smartphone system for self-management of schizophrenia. *Psychiatr Rehabil J*. 2013;36(4):289-296. doi:10.1037/prj0000019.
23. Bendixen RM, Fairman AD, Karavolis M, Sullivan C, Parmanto B. A user-centered approach: Understanding client and caregiver needs and preferences in the development of mhealth apps for self-management. *JMIR mHealth uHealth*. 2017;5(9):1-11. doi:10.2196/mhealth.7136.
24. Bevan Jones R, Thapar A, Rice F, et al. A web-based psychoeducational intervention for adolescent depression: design and development of MoodHwb. *J Med Internet Res*. 2018;5(1):1-19. doi:10.2196/mental.8894.
25. Biediger-friedman L, Crixell SH, Silva M, Markides BR, Smith KS. User-centered Design of a Texas WIC App: A Focus Group Investigation. *Am J Heal Behav*. 2016;40(4):461-471.
26. Birnie KA, Nguyen C, Do Amaral T, et al. A parent-science partnership to improve postsurgical pain management in young children: Co-development and usability testing of the Achy Penguin smartphone-based app. *Can J Pain = Rev Can La Douleur*. 2018;2(1):280-291. doi:10.1080/24740527.2018.1534543.
27. Birnie KA, Campbell F, Nguyen C, et al. iCanCope PostOp: User-Centered Design of a Smartphone-Based App for Self-Management of Postoperative Pain in Children and Adolescents. *JMIR Form Res*. 2019;3(2):e12028. doi:10.2196/12028.
28. Bishop FL, Greville-Harris M, Bostock J, et al. Using psychological theory and qualitative methods to develop a new evidence-based website about acupuncture for back pain. *Eur J Integr Med*. 2016;8(4):384-393. doi:10.1016/j.eujim.2016.05.006.
29. Bjerkan J, Hedlund M, Helleso R. Patients' contribution to the development of a web-based plan for integrated care - a participatory design study. *Informatics Heal Soc Care*. 2015;40(2):167-184. doi:10.3109/17538157.2014.907803.
30. Blijleven V, Koelemeijer K, Jaspers M. SEWA: A framework for sociotechnical analysis of electronic health record system workarounds. *Int J Med Inform*. 2019;125:71-78. doi:10.1016/j.ijmedinf.2019.02.012.
31. Bobin M, Anastassova M, Boukallel M, Ammi M. Design and Study of a Smart Cup for Monitoring the Arm and Hand Activity of Stroke Patients. *IEEE J Transl Eng Heal Med*. 2018;6:2100812. doi:10.1109/JTEHM.2018.2853553.

32. Boman IL, Persson AC, Bartfai A. First steps in designing an all-in-one ICT-based device for persons with cognitive impairment: Evaluation of the first mock-up. *BMC Geriatr*. 2016;16(61):1-11. doi:10.1186/s12877-016-0238-x.
33. Børøsund E, Mirkovic J, Clark MM, et al. A stress management app intervention for cancer survivors: Design, development, and usability testing. *J Med Internet Res*. 2018;2(2):1-16. doi:10.2196/formative.9954.
34. Bourazeri A, Stumpf S, Acm. Co-Designing Smart Home Technology with People with Dementia or Parkinson's Disease. *Nord '18 Proc 10th Nord Conf Human-Computer Interact*. 2018:609-621. doi:10.1145/3240167.3240197.
35. Brox E, Konstantinidis ST, Evertsen G. User-Centered Design of Serious Games for Older Adults Following 3 Years of Experience With Exergames for Seniors: A Study Design. *JMIR Serious Games*. 2017;5(1):1-14. doi:10.2196/games.6254.
36. Buitenweg DC, Bongers IL, van de Mheen D, van Oers HAM, van Nieuwenhuizen C. Cocreative Development of the QoL-ME: A Visual and Personalized Quality of Life Assessment App for People With Severe Mental Health Problems. *JMIR Ment Heal*. 2019;6(3). doi:10.2196/12378.
37. Cai RA, Beste D, Chaplin H, et al. Developing and evaluating JIApp: Acceptability and usability of a smartphone app system to improve self-management in young people with juvenile idiopathic arthritis. *JMIR mHealth uHealth*. 2017;5(8):1-19. doi:10.2196/mhealth.7229.
38. Calvillo-Arbizu J, Roa-Romero LM, Estudillo-Valderrama MA, et al. User-centred design for developing e-Health system for renal patients at home (AppNephro). *Int J Med Inform*. 2019;125(2019):47-54. doi:10.1016/j.ijmedinf.2019.02.007.
39. Casida JM, Aikens JE, Craddock H, Aldrich MW, Pagani FD. Development and Feasibility of Self-Management Application in Left-Ventricular Assist Devices. *ASAIO J*. 2018;64(2):159-167. doi:10.1097/MAT.0000000000000673.
40. Ceasar JN, Claudel SE, Andrews MR, et al. Community Engagement in the Development of an mHealth-Enabled Physical Activity and Cardiovascular Health Intervention (Step It Up): Pilot Focus Group Study. *JMIR Form Res*. 4AD;3(1):e10944. doi:10.2196/10944.
41. Chhoun P, Kaplan KC, Wieten C, et al. Using participatory methods to build an mHealth intervention for female entertainment workers in Cambodia: the development of the Mobile Link project. *mHealth*. 2019;5:24. doi:10.21037/mhealth.2019.07.02.
42. Cnossen IC, Van Uden-Kraan CF, Eerenstein SEJ, et al. A Participatory Design Approach to Develop a Web-Based Self-Care Program Supporting Early Rehabilitation among Patients after Total Laryngectomy. *Folia Phoniatr Logop*. 2016;2015(67):193-201. doi:10.1159/000441251.

43. Compagna D, Kohlbacher F. The limits of participatory technology development: The case of service robots in care facilities for older people. *Technol Forecast Soc Change*. 2015;93:19-31. doi:10.1016/j.techfore.2014.07.012.
44. Connelly K, Stein KF, Chaudry B, Trabold N. Development of an ecological momentary assessment mobile app for a low-literacy, Mexican American population to collect disordered eating behaviors. *JMIR Public Heal Surveill*. 2016;2(2):1-14. doi:10.2196/publichealth.5511.
45. Coons JC, Patel R, Coley KC, Empey PE. Design and testing of Medivate, a mobile app to achieve medication list portability via Fast Healthcare Interoperability Resources. *J Am Pharm Assoc*. 2019;59(2):S78-S85.e2. doi:10.1016/j.japh.2019.01.001.
46. Cordova D, Bauermeister JA, Fessler K, et al. A community-engaged approach to developing an mHealth HIV/STI and drug abuse preventive intervention for primary care: A qualitative study. *JMIR mHealth uHealth*. 2015;3(4):1-19. doi:10.2196/mhealth.4620.
47. Crehan C, Kesler E, Nambiar B, et al. The NeoTree application: developing an integrated mHealth solution to improve quality of newborn care and survival in a district hospital in Malawi. *BMJ Glob Heal*. 2019;4(1):e000860. doi:10.1136/bmjgh-2018-000860.
48. Cristancho-Lacroix V, Moulin F, Wrobel J, et al. A Web-Based Program for Informal Caregivers of Persons With Alzheimer’s Disease: An Iterative User-Centered Design. *JMIR Res Protoc*. 2014;3(3). doi:10.2196/resprot.3607.
49. Crosby LE, Ware RE, Goldstein A, et al. Development and evaluation of iManage: A self-management app co-designed by adolescents with sickle cell disease. *Pediatr Blood Cancer*. 2017;64(1):139-145. doi:10.1002/pbc.26177.
50. Curtis KE, Lahiri S, Brown KE. Targeting Parents for Childhood Weight Management: Development of a Theory-Driven and User-Centered Healthy Eating App. *JMIR mHealth uHealth*. 2015;3(2):1-18. doi:10.2196/mhealth.3857.
51. Dack C, Ross J, Stevenson F, et al. A digital self-management intervention for adults with type 2 diabetes: Combining theory, data and participatory design to develop HeLP-Diabetes. *Internet Interv*. 2019;17:100241. doi:https://doi.org/10.1016/j.invent.2019.100241.
52. Danbjørg DB, Villadsen A, Gill E, Rothmann MJ, Clemensen J. Usage of an Exercise App in the Care for People With Osteoarthritis: User-Driven Exploratory Study. *JMIR mHealth uHealth*. 2018;6(1):e11-e11. doi:10.2196/mhealth.7734.
53. Davis SR, Peters D, Calvo RA, Sawyer SM, Foster JM, Smith L. “Kiss myAsthma”: Using a participatory design approach to develop a self-management app with young people with asthma. *J Asthma*. 2018;55(9):1018-1027. doi:10.1080/02770903.2017.1388391.
54. De la Vega R, Roset Mayals R, Castarlenas E, Sánchez-Rodríguez E, Solé E, Miró J. Development and Testing of Painometer: A Smartphone App to Assess Pain Intensity. *J Pain*. 2014;15(10). doi:10.1016/j.jpain.2014.04.009.

55. de Souza S, Galloway J, Simpson C, et al. Patient involvement in rheumatology outpatient service design and delivery: a case study. *Heal Expect an Int J public Particip Heal care Heal policy*. 2017;20(3):508-518. doi:10.1111/hex.12478.
56. Deighan C, Michalova L, Pagliari C, Elliott J, Taylor L, Ranaldi H. The Digital Heart Manual: A pilot study of an innovative cardiac rehabilitation programme developed for and with users. *Patient Educ Couns*. 2017;100(8):1598-1607. doi:10.1016/j.pec.2017.03.014.
57. den Bakker CM, Schaafsma FG, Van Der Meij E, et al. Electronic Health Program to Empower Patients in Returning to Normal Activities After General Surgical and Gynecological Procedures: Intervention Mapping as a Useful Method for Further Development. *J Med Internet Res*. 6AD;21(2):e9938. doi:10.2196/jmir.9938.
58. Dimeff LA, Jobes DA, Chalker SA, et al. A novel engagement of suicidality in the emergency department: Virtual Collaborative Assessment and Management of Suicidality. *Gen Hosp Psychiatry*. 2020;63(May):119-126. doi:10.1016/j.genhosppsych.2018.05.005.
59. Dirin M, Dirin A, Laine TH, Acm. User-Centered Design of a Context-Aware Nurse Assistant (CANA) at Finnish Elderly Houses. *Proc 9th Int Conf Ubiquitous Inf Manag Commun*. 2015. doi:10.1145/2701126.2701225.
60. Doarn CR, Vonder Meulen MB, Pallerla H, et al. Challenges in the Development of e-Quit worRx: An iPad App for Smoking Cessation Counseling and Shared Decision Making in Primary Care. *JMIR Form Res*. 29AD;3(1):e11300. doi:10.2196/11300.
61. Domańska B, Stumpp O, Poon S, Oray S, Mountian I, Pichon C. Using Patient Feedback to Optimize the Design of a Certolizumab Pegol Electromechanical Self-Injection Device: Insights from Human Factors Studies. *Adv Ther*. 2018;35(1):100-115. doi:10.1007/s12325-017-0645-1.
62. Dorrington P, Wilkinson C, Tasker L, Walters A. User-Centered Design Method for the Design of Assistive Switch Devices to Improve User Experience, Accessibility, and Independence. *J Usability Stud*. 2016;11(2):66-82.
63. Dowd AJ, Jackson C, Tang KTY, Nielsen D, Clarkin DH, Culos-Reed SN. MyHealthyGut: development of a theory-based self-regulatory app to effectively manage celiac disease. *mHealth*. 2018;4(19):1-16. doi:10.21037/mhealth.2018.05.05.
64. Duval J, Segura EM, Kurniawan SH. SpokeIt: A Co-Created Speech Therapy Experience. *Ext Abstr 2018 CHI Conf Hum Factors Comput Syst*. 2018.
65. Dworkin M, Chakraborty A, Lee S, et al. A realistic talking human embodied agent mobile phone intervention to promote HIV medication adherence and retention in care in young HIV-positive African American men who have sex with men: Qualitative study. *JMIR mHealth uHealth*. 2018;6(7):1-11. doi:10.2196/10211.

66. Dykes PC, Stade D, Chang F, et al. Participatory Design and Development of a Patient-centered Toolkit to Engage Hospitalized Patients and Care Partners in their Plan of Care. *AMIA Annu Symp Proc*. 2014;2014:486-495.

67. Easton K, Potter S, Bec R, et al. A Virtual Agent to Support Individuals Living With Physical and Mental Comorbidities: Co-Design and Acceptability Testing. *J Med Internet Res*. 30AD;21(5):e12996. doi:10.2196/12996.

68. Edwards EA, Caton H, Lumsden J, et al. Creating a theoretically grounded, gamified health app: Lessons from developing the cigbreak smoking cessation mobile phone game. *JMIR Serious Games*. 2018;6(4):1-20. doi:10.2196/10252.

69. Ehrler F, Lovis C, Blondon K. A Mobile Phone App for Bedside Nursing Care: Design and Development Using an Adapted Software Development Life Cycle Model. *JMIR Mhealth Uhealth*. 11AD;7(4):e12551. doi:10.2196/12551.

70. Elbæk L, Hjort A, Khalid S. Designing an interactive wall for movement: For-and with-intellectual disabled people. In: *Proceedings of the European Conference on Games-Based Learning*. Vol 12. ; 2018:62-71.

71. Elsbernd A, Hjerming M, Visler C, et al. Using Cocreation in the Process of Designing a Smartphone App for Adolescents and Young Adults with Cancer: Prototype Development Study. *JMIR Form Res*. 2018;2(2):1-9. doi:10.2196/formative.9842.

72. Escalada-Hernández P, Soto Ruiz N, San Martín-Rodríguez L, et al. Design and evaluation of a prototype of augmented reality applied to medical devices. *Int J Med Inf*. 2019;128(2019):87-92. doi:10.1016/j.ijmedinf.2019.05.004.

73. Ettinger KM, Pharaoh H, Buckman RY, Conradie H, Karlen W. Building quality mHealth for low resource settings. *J Med Eng Technol*. 2016;40(7-8):431-443. doi:10.1080/03091902.2016.1213906.

74. Fager SK, Sorenson T, Butte S, Nelson A, Banerjee N, Robucci R. Integrating end-user feedback in the concept stage of development of a novel sensor access system for environmental control. *Disabil Rehabil Assist Technol*. 2018;13(4):366-372. doi:10.1080/17483107.2017.1328615.

75. Feldner HA, Logan SW, Galloway JC. Mobility in pictures: a participatory photovoice narrative study exploring powered mobility provision for children and families. *Disabil Rehabil Assist Technol*. 2019;14(3):301-311. doi:10.1080/17483107.2018.1447606.

76. Fernon A, Nguyen A, Baysari M, Day R. A user-centred approach to designing an etool for gout management. *Stud Health Technol Inform*. 2016;227:28-33. doi:10.3233/978-1-61499-666-8-28.

77. Fledderus M, Schreurs KM, Bohlmeijer ET, Vollenbroek-Hutten MM. Development and Pilot Evaluation of an Online Relapse-Prevention Program Based on Acceptance and Commitment

Therapy for Chronic Pain Patients. *JMIR Hum Factors*. 2015;2(1):1-12. doi:10.2196/humanfactors.3302.

78. Fortuna KL, Naslund JA, Aschbrenner KA, et al. Text message exchanges between older adults with serious mental illness and older certified peer specialists in a smartphone-supported self-management intervention. *Psychiatr Rehabil J*. 2019;42(1):57-63.

79. Gabrielli S, Dianti M, Maimone R, et al. Design of a mobile app for nutrition education (Trec-lifestyle) and formative evaluation with families of overweight children. *JMIR mHealth uHealth*. 2017;5(4):1-13. doi:10.2196/mhealth.7080.

80. Gardsten C, Mörtberg C, Blomqvist K. Designing an ICT self-management service: suggestions from persons with type 2 diabetes. *Health Technol (Berl)*. 2017;7(2):197-206. doi:10.1007/s12553-016-0176-9.

81. Garne Holm K, Brødsgaard A, Zachariassen G, Smith AC, Clemensen J. Participatory design methods for the development of a clinical telehealth service for neonatal homecare. *SAGE open Med*. 2017;5:2050312117731252-2050312117731252. doi:10.1177/2050312117731252.

82. Garzo A, Silva PA, Garay-Vitoria N, et al. Design and development of a gait training system for Parkinson’s disease. *PLoS One*. 2018;13(11):1-30. doi:10.1371/journal.pone.0207136.

83. Geraghty AW, Muñoz RF, Yardley L, Mc Sharry J, Little P, Moore M. Developing an Unguided Internet-Delivered Intervention for Emotional Distress in Primary Care Patients: Applying Common Factor and Person-Based Approaches. *JMIR Ment Heal*. 2016;3(4):1-14. doi:10.2196/mental.5845.

84. Ghysels R, Vanroye E, Westhovens M, Spooren A. A tool to enhance occupational therapy reasoning from ICF perspective: The Hasselt Occupational Performance Profile (H-OPP). *Scand J Occup Ther*. 2017;24(2):126-135. doi:10.1080/11038128.2016.1217925.

85. Gilbert M, Haag D, Hottes TS, et al. Get Checked… Where? The Development of a Comprehensive, Integrated Internet-Based Testing Program for Sexually Transmitted and Blood-Borne Infections in British Columbia, Canada. *JMIR Res Protoc*. 2016;5(3):1-18. doi:10.2196/resprot.6293.

86. Gill R, Ogilvie G, Norman W V., Fitzsimmons B, Maher C, Renner R. Feasibility and Acceptability of a Mobile Technology Intervention to Support Postabortion Care in British Columbia: Phase I. *J Med Internet Res*. 29AD;21(5):e13387. doi:10.2196/13387.

87. Ginsburg AS, Delarosa J, Brunette W, et al. mPneumonia: Development of an Innovative mHealth Application for Diagnosing and Treating Childhood Pneumonia and Other Childhood Illnesses in Low-Resource Settings. *PLoS One*. 2015;10(10):e0139625. doi:10.1371/journal.pone.0139625.

88. Giordanengo A, Ozturk P, Hansen AH, Årsand E, Grøttland A, Hartvigsen G. Design and development of a context-aware knowledge-based module for identifying relevant information

and information gaps in patients with type 1 diabetes self-collected health data. *J Med Internet Res*. 2018;3(3):1-18. doi:10.2196/10431.
89. Gogovor A, Visca R, Auger C, et al. Informing the development of an Internet-based chronic pain self-management program. *Int J Med Inform*. 2017;97:109-119. doi:10.1016/j.ijmedinf.2016.10.005.
90. Goncu-Berk G, Topcuoglu N. A Healthcare Wearable for Chronic Pain Management. Design of a Smart Glove for Rheumatoid Arthritis. *Des J*. 2017;20(12th International Conference of the European-Academy-of-Design (EAD)):S1978-S1988. doi:10.1080/14606925.2017.1352717.
91. Goodwin J, Cummins J, Behan L, O'Brien SM. Development of a mental health smartphone app: perspectives of mental health service users. *J Ment Heal*. 2016;25(5):434-440. doi:10.3109/09638237.2015.1124392.
92. Gordon JS, Armin JS, Cunningham JK, Muramoto ML, Christiansen SM, Jacobs TA. Lessons learned in the development and evaluation of RxCoach™, an mHealth app to increase tobacco cessation medication adherence. *Patient Educ Couns*. 2017;100(4):720-727. doi:10.1016/j.pec.2016.11.003.
93. Gordon M, Henderson R, Holmes JH, Wolters MK, Bennett IM. Participatory design of eHealth solutions for women from vulnerable populations with perinatal depression. *J Am Med Informatics Assoc*. 2016;23(1):105-109. doi:10.1093/jamia/ocv109.
94. Gracey LE, Zan S, Gracz J, et al. Use of user-centered design to create a smartphone application for patient-reported outcomes in atopic dermatitis. *NPJ Digit Med*. 2018;1:33. doi:10.1038/s41746-018-0042-4.
95. Grainger R, Townsley H, Langlotz T, Taylor W. Patient-Clinician Co-Design Co-Participation in Design of an App for Rheumatoid Arthritis Management via Telehealth Yields an App with High Usability and Acceptance. *Stud Health Technol Inform*. 2017;245:1223.
96. Grasaas E, Fegran L, Helseth S, et al. iCanCope With Pain: Cultural Adaptation and Usability Testing of a Self-Management App for Adolescents With Persistent Pain in Norway. *JMIR Res Protoc*. 2019;8(6):e12940. doi:10.2196/12940.
97. Gray TG, Moores KL, James E, Connor ME, Jones GL, Radley SC. Development and initial validation of an electronic personal assessment questionnaire for menstrual, pelvic pain and gynaecological hormonal disorders (ePAQ-MPH). *Eur J Obstet Gynecol Reprod Biol*. 2019;238:148-156. doi:10.1016/j.ejogrb.2019.05.024.
98. Grindell C, Mawson S, Gerrish K, Parker S, Bissell P. Exploring the acceptability and usability of a novel social innovation to encourage physical activity: The iStep prototype. *Health Soc Care Community*. 2019;27(2):383-391. doi:10.1111/hsc.12656.

99. Groussard P-Y, Pigot H, Giroux S. From conception to evaluation of mobile services for people with head injury: A participatory design perspective. *Neuropsychol Rehabil*. 2018;28(5):667-688. doi:10.1080/09602011.2015.1117499.

100. Guédon ACP, Wauben LSGL, de Korne DF, Overvelde M, Dankelman J, van den Dobbelsteen JJ. A RFID specific participatory design approach to support design and implementation of real-time location systems in the operating room. *J Med Syst*. 2015;39(1):168. doi:10.1007/s10916-014-0168-0.

101. Han N, Han SH, Chu H, et al. Service design oriented multidisciplinary collaborative team care service model development for resolving drug related problems. *PLoS One*. 2018;13(9):e0201705. doi:10.1371/journal.pone.0201705.

102. Hanschke V, Manataki A, Alexandru CA, et al. Designing a Social Machine for the Heart Manual Service. VanDenBroek EL, Fred A, Gamboa H, Vaz M, eds. *Proc 10th Int Jt Conf Biomed Eng Syst Technol (BIOSTEC 2017)*. 2017;(10th International Joint Conference on Biomedical Engineering Systems and Technologies):435-440. doi:10.5220/0006249004350440.

103. Hanschke V, Manataki A, Alexandru C, et al. *Designing a Social Machine for the Heart Manual Service*.; 2017. doi:10.5220/0006249004350440.

104. Hardinge M, Rutter H, Velardo C, et al. Using a mobile health application to support self-management in chronic obstructive pulmonary disease: a six-month cohort study. *Hardinge, M, Rutter, H, Velardo, C, Shah, S A, Williams, V, Tarassenko, L, Farmer, A (2015) Using a Mob Heal Appl to Support self-management chronic Obstr Pulm Dis a six-month cohort study BMC Med informatics*. 2015;15(46). doi:10.1186/s12911-015-0171-5.

105. Hardy A, Wojdecka A, West J, et al. How Inclusive, User-Centered Design Research Can Improve Psychological Therapies for Psychosis: Development of SlowMo. *JMIR Ment Heal*. 2018;5(4). doi:10.2196/11222.

106. Harricharan M, Gemen R, Celemín LF, et al. Integrating mobile technology with routine dietetic practice: the case of myPace for weight management. *Proc Nutr Soc*. 2015;74(2):125-129. doi:10.1017/S0029665115000105.

107. Harte R, Quinlan LR, Glynn L, et al. Human-centered design study: Enhancing the usability of a mobile phone app in an integrated falls risk detection system for use by older adult users. *JMIR mHealth uHealth*. 2017;5(5):1-24. doi:10.2196/mhealth.7046.

108. Hasin DS, Aharonovich E, Greenstein E. HealthCall for the smartphone: technology enhancement of brief intervention in HIV alcohol dependent patients. *Addict Sci Clin Pract*. 2014;9(1):5. doi:10.1186/1940-0640-9-5.

109. Hauffman A, Alfonsson S, Mattsson S, et al. The Development of a Nurse-Led Internet-Based Learning and Self-care Program for Cancer Patients With Symptoms of Anxiety and

Depression-A Part of U-CARE. *Cancer Nurs*. 2017;40(5):E9-E16. doi:10.1097/NCC.0000000000000402.

110. Haynes SC, Kim KK. A mobile system for the improvement of heart failure management: Evaluation of a prototype. *AMIA . Annu Symp proceedings AMIA Symp*. 2018;2017:839-848. https://pubmed.ncbi.nlm.nih.gov/29854150.
111. Haynes S, Kim KK. A Mobile Care Coordination System for the Management of Complex Chronic Disease. *Stud Health Technol Inform*. 2016;225:505-509.
112. Heynsbergh N, Heckel L, Botti M, et al. Development of a Smartphone App for Informal Carers of People With Cancer: Processes and Learnings. *JMIR Form Res*. 2019;3(2):e10990. doi:10.2196/10990.
113. Hill AJ, Breslin HM. Refining an Asynchronous Telerehabilitation Platform for Speech-Language Pathology: Engaging End-Users in the Process. *Front Hum Neurosci*. 2016;10:640. doi:10.3389/fnhum.2016.00640.
114. Ho G, Ueda K, Houben RFA, et al. Metabolic Diet App Suite for inborn errors of amino acid metabolism. *Mol Genet Metab*. 2016;117(3):322-327. doi:10.1016/j.ymgme.2015.12.007.
115. Hochstenbach LMJ, Courtens AM, Zwakhalen SMG, Vermeulen J, van Kleef M, de Witte LP. Co-creative development of an eHealth nursing intervention: Self-management support for outpatients with cancer pain. *Appl Nurs Res*. 2017;36:1-8. doi:10.1016/j.apnr.2017.03.004.
116. Hodson E, Dadashi N, Delgado R, Chisholm C, Sgrignoli R, Swaine R. Co-design in mental health; Mellow: a self-help holistic crisis planning mobile application by youth, for youth. *Des J*. 2019;22:1529-1542. doi:10.1080/14606925.2019.1594975.
117. Hohenstein J, O'Dell D, Murnane EL, Lu Z, Erickson D, Gay G. Enhancing the Usability of an Optical Reader System to Support Point-of-Care Rapid Diagnostic Testing: An Iterative Design Approach. *JMIR Hum Factors*. 2017;4(4):1-13. doi:10.2196/humanfactors.8621.
118. Holtz BE, Murray KM, Hershey DD, et al. Developing a patient-centered mhealth app: A tool for adolescents with type 1 diabetes and their parents. *JMIR mHealth uHealth*. 2017;5(4):1-11. doi:10.2196/mhealth.6654.
119. Honary M, Fisher NR, McNaney R, Lobban F. A Web-Based Intervention for Relatives of People Experiencing Psychosis or Bipolar Disorder: Design Study Using a User-Centered Approach. *JMIR Ment Heal*. 2018;5(4):1-15. doi:10.2196/11473.
120. Houzé de l'Aulnoit A, Boudet S, Génin M, et al. Development of a Smart Mobile Data Module for Fetal Monitoring in E-Healthcare. *J Med Syst*. 2018;42(5):83. doi:10.1007/s10916-018-0938-1.
121. Howarth A, Quesada J, Donnelly T, Mills PR. The development of 'Make One Small Change': an e-health intervention for the workplace developed using the Person-Based Approach. *Digit Heal*. 2019;5:1-11. doi:10.1177/2055207619852856.

122. Hsieh KL, Fanning JT, Rogers WA, Wood TA, Sosnoff JJ. A Fall Risk mHealth App for Older Adults: Development and Usability Study. *JMIR aging*. 2018;1(2):e11569-e11569. doi:10.2196/11569.

123. Huberty J, Rowedder L, Hekler E, et al. Development and design of an intervention to improve physical activity in pregnant women using Text4baby. *Transl Behav Med*. 2016;6(2):285-294. doi:10.1007/s13142-015-0339-7.

124. Islind AS, Lindroth T, Lundin J, Steineck G. Co-designing a digital platform with boundary objects: bringing together heterogeneous users in healthcare. *Health Technol (Berl)*. 2019;9(4):425-438. doi:10.1007/s12553-019-00332-5.

125. Jaensson M, Dahlberg K, Eriksson M, Gronlund A, Nilsson U. The Development of the Recovery Assessments by Phone Points (RAPP): A Mobile Phone App for Postoperative Recovery Monitoring and Assessment. *JMIR mHealth uHealth*. 2015;3(3). doi:10.2196/mhealth.4649.

126. Jalil S, Myers T, Atkinson I, Soden M. Complementing a Clinical Trial With Human-Computer Interaction: Patients' User Experience With Telehealth. *JMIR Hum Factors*. 2019;6(2):e9481. doi:10.2196/humanfactors.9481.

127. Jamin G, Luyten T, Delsing R, Braun S. The process of co-creating the interface for VENSTER, an interactive artwork for nursing home residents with dementia. *Disabil Rehabil Assist Technol*. 2018;13(8):809-818. doi:10.1080/17483107.2017.1385102.

128. Jibb LA, Cafazzo JA, Nathan PC, et al. Development of a mHealth Real-Time Pain Self-Management App for Adolescents With Cancer: An Iterative Usability Testing Study [Formula: see text]. *J Pediatr Oncol Nurs Off J Assoc Pediatr Oncol Nurses*. 2017;34(4):283-294. doi:10.1177/1043454217697022.

129. Jie LJ, Jamin G, Smit K, Beurskens A, Braun S. Design of the user interface for "Stappy", a sensor-feedback system to facilitate walking in people after stroke: a user-centred approach. *Disabil Rehabil Assist Technol*. 2020;15(8):959-967. doi:10.1080/17483107.2019.1629654.

130. Joe J, Hall A, Chi NC, Thompson H, Demiris G. IT-based wellness tools for older adults: Design concepts and feedback. *Informatics Heal Soc Care*. 2018;43(2):142-158. doi:10.1080/17538157.2017.1290637.

131. Joensson K, Melholt C, Hansen J, et al. Listening to the patients: using participatory design in the development of a cardiac telerehabilitation web portal. *mHealth*. 2019;5:33. doi:10.21037/mhealth.2019.08.06.

132. Joshi A, Amadi C, Schumer H, Galitzdorfer L, Gaba A. A human centered approach to design a diet app for patients with metabolic syndrome. *mHealth*. 2019;5:43. doi:10.21037/mhealth.2019.08.13.

133. Kabeza CB, Harst L, Schwarz PEH, Timpel P. Assessment of Rwandan diabetic patients' needs and expectations to develop their first diabetes self-management smartphone application (Kir'App). *Ther Adv Endocrinol Metab*. 2019;10:2042018819845318.
134. Kildea J, Battista J, Cabral B, et al. Design and Development of a Person-Centered Patient Portal Using Participatory Stakeholder Co-Design. *J Med Internet Res*. 2019;21(2). doi:10.2196/11371.
135. Kim B, Baldwin J, Lukasiewicz K, et al. Development of Tablet Device App for Parkinson's Disease Patients' Continuous Self-Monitoring and Management. *Neurosci Biomed Eng*. 2015;3. doi:10.2174/2213385203666150722234734.
136. Kim KK, Logan HC, Young E, Sabee CM. Youth-centered design and usage results of the iN Touch mobile self-management program for overweight/obesity. *Pers Ubiquitous Comput*. 2015;19(1):59-68. doi:10.1007/s00779-014-0808-x.
137. Kip H, Kelders SM, Weerink K, et al. Identifying the Added Value of Virtual Reality for Treatment in Forensic Mental Health: A Scenario-Based, Qualitative Approach. *Front Psychol*. 2019;10:406. doi:10.3389/fpsyg.2019.00406.
138. Knight-Agarwal C, Davis DL, Williams L, Davey R, Cox R, Clarke A. Development and Pilot Testing of the Eating4two Mobile Phone App to Monitor Gestational Weight Gain. *JMIR mHealth uHealth*. 2015;3(2):1-11. doi:10.2196/mhealth.4071.
139. Kohlstadt IC, Anderson Steeves ET, Rice K, Gittelsohn J, Summerfield LM, Gadhoke P. Youth peers put the "invent" into NutriBee's online intervention. *Nutr J*. 2015;14(1):1-8. doi:10.1186/s12937-015-0031-2.
140. Krishnamurti T, Davis AL, Wong-Parodi G, Fischhoff B, Sadovsky Y, Simhan HN. Development and testing of the myhealthypregnancy app: A behavioral decision research-based tool for assessing and communicating pregnancy risk. *JMIR mHealth uHealth*. 2017;5(4):1-11. doi:10.2196/mhealth.7036.
141. Kristiansen AM, Svanholm JR, Schjodt I, Jensen KM, Silen C, Karlgren K. Patients with heart failure as co-designers of an educational website: implications for medical education. *Int J Med Educ*. 2017;8:47-58. doi:10.5116/ijme.5898.309e.
142. Lau Y, Cheng LJ, Chi C, et al. Development of a healthy lifestyle mobile app for overweight pregnant women: Qualitative study. *JMIR mHealth uHealth*. 2018;6(4):1-17. doi:10.2196/mhealth.9718.
143. Lee HR, Sabanovic S, Chang WL, et al. Steps Toward Participatory Design of Social Robots: Mutual Learning with Older Adults with Depression. *2017 12th ACM/IEEE Int Conf Human-Robot Interact*. 2017:244-253. doi:10.1145/2909824.3020237.
144. Leorin C, Stella E, Nugent C, et al. The Value of Including People with Dementia in the Co-Design of Personalized eHealth Technologies. *Dement Geriatr Cogn Disord*. 2019;47(3):164-175. doi:10.1159/000497804.

145. Lipson-Smith R, White F, White A, et al. Co-Design of a Consultation Audio-Recording Mobile App for People With Cancer: The SecondEars App. *JMIR Form Res*. 2019;3(1):e11111. doi:10.2196/11111.
146. Liu Y, Geng Z, Wu F, Yuan C. Developing "Information Assistant": A Smartphone Application to Meet the Personalized Information Needs of Women with Breast Cancer. *Stud Health Technol Inform*. 2017;245:156-160.
147. Lwin MO, Vijaykumar S, Rathnayake VS, et al. A social media mHealth solution to address the needs of dengue prevention and management in Sri Lanka. *J Med Internet Res*. 2016;18(7):1-15. doi:10.2196/jmir.4657.
148. Maher M, Kaziunas E, Ackerman M, et al. User-Centered Design Groups to Engage Patients and Caregivers with a Personalized Health Information Technology Tool. *Biol blood marrow Transplant J Am Soc Blood Marrow Transplant*. 2016;22(2):349-358. doi:10.1016/j.bbmt.2015.08.032.
149. Marent B, Henwood F, Darking M. Ambivalence in digital health: Co-designing an mHealth platform for HIV care. *Soc Sci Med*. 2018;215:133-141. doi:10.1016/j.socscimed.2018.09.003.
150. Marent B, Henwood F, Darking M, et al. Development of an mHealth platform for HIV care: Gathering user perspectives through co-design workshops and interviews. *JMIR mHealth uHealth*. 2018;6(10):1-15. doi:10.2196/mhealth.9856.
151. Marien S, Legr, Ramdoyal R, et al. A User-Centered design and usability testing of a web-based medication reconciliation application integrated in an eHealth network. *Int J Med Inf*. 2019;126(June 2019):138-146. doi:10.1016/j.ijmedinf.2019.03.013.
152. Marsac ML, Winston FK, Hildenbrand AK, et al. Systematic, theoretically grounded development and feasibility testing of an innovative, preventive web-based game for children exposed to acute trauma. *Clin Pract Pediatr Psychol*. 2015;3(1):12-24. doi:10.1037/cpp0000080.
153. Martinez A, Mora R, López G, et al. Design and Evaluation of a Personalized Cancer Treatment System Using Human-Computer Interaction Techniques. Rocha Á, Correia AM, Adeli H, Reis LP, Mendonça Teixeira M, eds. *New Adv Inf Syst Technol Adv Intell Syst Comput*. 2016;444:717-727.
154. Martinez W, Threatt AL, Trent Rosenbloom S, Wallston KA, Hickson GB, Elasy TA. A patient-facing diabetes dashboard embedded in a patient web portal: Design sprint and usability testing. *J Med Internet Res*. 2018;5(3):1-17. doi:10.2196/humanfactors.9569.
155. Mateo KF, Berner NB, Ricci NL, et al. Development of a 5As-based technology-assisted weight management intervention for veterans in primary care. *BMC Health Serv Res*. 2018;18(47):1-14. doi:10.1186/s12913-018-2834-2.

156. Materia FT, Smyth JM, Heron KE, et al. Preconceptional health behavior change in women with overweight and obesity: prototype for SMART strong healthy women intervention. *mHealth*. 2018;4(24):1-11. doi:10.21037/mhealth.2018.06.06.
157. Mattie J, Wong A, Leland D, Borisoff J. End user evaluation of a Kneeling Wheelchair with "on the fly" adjustable seating functions. *Disabil Rehabil Assist Technol*. 2019;14(6):543-554. doi:10.1080/17483107.2018.1462861.
158. Mawson S, Nasr N, Parker J, Zheng H, Davies R, Mountain G. Developing a personalised self-management system for post stroke rehabilitation; utilising a user-centred design methodology. *Disabil Rehabil Assist Technol*. 2014;9(6):521-528. doi:10.3109/17483107.2013.840863.
159. McClelland GT, Fitzgerald M. A participatory mobile application (app) development project with mental health service users and clinicians. *Health Educ J*. 2018;77(7):815-827. doi:10.1177/0017896918773790.
160. McDonald L, Glen FC, Taylor DJ, Crabb DP. Self-Monitoring Symptoms in Glaucoma: A Feasibility Study of a Web-Based Diary Tool. *J Ophthalmol*. 2017;2017. doi:10.1155/2017/8452840.
161. McGrath SP, McGrath ML, Bastola D. Developing a concussion assessment mHealth app for certified Athletic Trainers. *AMIA . Annu Symp proceedings AMIA Symp*. 2017;2017:1282-1291.
162. Michalak EE, Morton E, Barnes SJ, Hole R, Murray G. Supporting Self-Management in Bipolar Disorder: Mixed-Methods Knowledge Translation Study. *JMIR Ment Heal*. 2019;6(4):e13493. doi:10.2196/13493.
163. Middelweerd A, te Velde SJ, Mollee JS, Klein MC, Brug J. App-Based Intervention Combining Evidence-Based Behavior Change Techniques With a Model-Based Reasoning System to Promote Physical Activity Among Young Adults (Active2Gether): Descriptive Study of the Development and Content. *JMIR Res Protoc*. 2018;7(12):1-15. doi:10.2196/resprot.7169.
164. Milward J, Deluca P, Drummond C, Watson R, Dunne J, Kimergard A. Usability Testing of the BRANCH Smartphone App Designed to Reduce Harmful Drinking in Young Adults. *JMIR mHealth uHealth*. 2017;5(8). doi:10.2196/mhealth.7836.
165. Milward J, Khadjesari Z, Fincham-Campbell S, Deluca P, Watson R, Drummond C. User preferences for content, features, and style for an app to reduce harmful drinking in young adults: Analysis of user feedback in app stores and focus group interviews. *JMIR mHealth uHealth*. 2016;4(2):1-15. doi:10.2196/mhealth.5242.
166. Mohamad Marzuki MF, Yaacob NA, Bin Yaacob NM, Abu Hassan MR, Ahmad SB. Usable Mobile App for Community Education on Colorectal Cancer: Development Process and Usability Study. *JMIR Hum Factors*. 2019;6(2):e12103. doi:10.2196/12103.

167. Morrison CF, Szulczewski L, Strahlendorf LF, Lane JB, Mullins LL, Pai ALH. Designing Technology to Address Parent Uncertainty in Childhood Cancer. *ANS Adv Nurs Sci*. 2016;39(1):15-25. doi:10.1097/ANS.0000000000000100.

168. Mortenson W Ben, Singh G, MacGillivray M, et al. Development of a Self-Management App for People with Spinal Cord Injury. *J Med Syst*. 2019;43(6):145. doi:10.1007/s10916-019-1273-x.

169. Naegele L, Ryöppy M, Wilde D. PDFi: participatory design fiction with vulnerable users. *Nord 2018 Revisiting Life Cycle - Proc 10th Nord Conf Human-Computer Interact*. September 2018:819-831. doi:10.1145/3240167.3240272.

170. Neubeck L, Coorey G, Peiris D, et al. Development of an integrated e-health tool for people with, or at high risk of, cardiovascular disease: The Consumer Navigation of Electronic Cardiovascular Tools (CONNECT) web application. *Int J Med Inform*. 2016;96:24-37. doi:10.1016/j.ijmedinf.2016.01.009.

171. Newby K, Crutzen R, Brown K, et al. An Intervention to Increase Condom Use Among Users of Chlamydia Self-Sampling Websites (Wrapped): Intervention Mapping and Think-Aloud Study. *JMIR Form Res*. 2019;3(2):e11242. doi:10.2196/11242.

172. Nguyen AD, Frensham LJ, Wong MXC, et al. Mhealth app patient testing and review of educational materials designed for self-management of gout patients: Descriptive qualitative studies. *JMIR mHealth uHealth*. 2018;6(10):1-12. doi:10.2196/mhealth.9811.

173. Ning P, Gao D, Cheng P, et al. Needs Analysis for a Parenting App to Prevent Unintentional Injury in Newborn Babies and Toddlers: Focus Group and Survey Study Among Chinese Caregivers. *JMIR Mhealth Uhealth*. 2019;7(4):e11957. doi:10.2196/11957.

174. Nitsch M, Dimopoulos CN, Flaschberger E, et al. A guided online and mobile self-help program for individuals with eating disorders: An iterative engagement and usability study. *J Med Internet Res*. 2016;18(1):1-11. doi:10.2196/JMIR.4972.

175. Nkoy FL, Hofmann MG, Stone BL, et al. Information needs for designing a home monitoring system for children with medical complexity. *Int J Med Inform*. 2019;122(2019):7-12. doi:10.1016/j.ijmedinf.2018.11.011.

176. Noergaard B, Sandvei M, Rottmann N, et al. Development of a Web-Based Health Care Intervention for Patients With Heart Disease: Lessons Learned From a Participatory Design Study. *JMIR Res Protoc*. 2017;6(5):1-8. doi:10.2196/resprot.7084.

177. O'Brien N, Heaven B, Teal G, et al. Integrating evidence from systematic reviews, qualitative research, and expert knowledge using co-design techniques to develop a web-based intervention for people in the retirement transition. *J Med Internet Res*. 2016;18(8):1-19. doi:10.2196/jmir.5790.

178. O'Reilly SL, Laws R. Health-e mums: Evaluating a smartphone app design for diabetes prevention in women with previous gestational diabetes. *Nutr Diet*. 2019;76(5):507-514. doi:10.1111/1747-0080.12461.
179. Ogden J, Maxwell H, Wong A. Development and feasibility study of an app (Ladle) for weight loss and behaviour change. *PeerJ*. 2019;7(2019):e6907. doi:10.7717/peerj.6907.
180. Ogrin R, Viswanathan R, Aylen T, Wallace F, Scott J, Kumar D. Co-design of an evidence-based health education diabetes foot app to prevent serious foot complications: a feasibility study. *Pract Diabetes*. 2018;35:203-209d. doi:10.1002/pdi.2197.
181. Olney CM, Vos-Draper T, Egginton J, et al. Development of a comprehensive mobile assessment of pressure (CMAP) system for pressure injury prevention for veterans with spinal cord injury. *J Spinal Cord Med*. 2019;42(6):1-10. doi:10.1080/10790268.2019.1570437.
182. Ortiz-Fernandez L, Garcia-Fernández R, Alava-Menica A, et al. Patients as codesigners of STARR system prototype. *PervasiveHealth'19 13th Int Conf Pervasive Comput Technol Healthc*. March 2019:422-425. doi:10.1145/1234567890?
183. Ospina-Pinillos L, Davenport TA, Ricci CS, Milton AC, Scott EM, Hickie IB. Developing a mental health eclinic to improve access to and quality of mental health care for young people: Using participatory design as research methodologies. *J Med Internet Res*. 2018;20(5):1-18. doi:10.2196/JMIR.9716.
184. Oulton K, Oldrieve N, Bayliss J, et al. Using participatory and creative research methods to develop and pilot an informative game for preparing children for blood tests. *Arts Health*. 2018;10(3):227-240. doi:10.1080/17533015.2017.1392329.
185. Paay J, Nielsen H, Larsen H, Kjeldskov J, Acm. Happy Bits: Interactive Technologies Helping Young Adults With Low Self-Esteem. *Nord '18 Proc 10th Nord Conf Human-Computer Interact*. 2018:584-596. doi:10.1145/3240167.3240180.
186. Page R. Integrating The User Into The Formative Stages of Medical Device Usability Design: A Speculative and Collaborative Approach. *Des J*. 2018;21(6):863-872. doi:10.1080/14606925.2018.1525830.
187. Panek P, Fazekas G, Lüftenegger T, et al. On the Prototyping of an ICT-Enhanced Toilet System for Assisting Older Persons Living Independently and Safely at Home. *Stud Health Technol Inform*. 2017;236:176-183.
188. Paulino T, Muñoz J, Bermudez S, Cameirão MS. Design of an Integrative System for Configurable Exergames Targeting the Senior Population BT - Human Systems Engineering and Design. In: Ahram T, Karwowski W, Taiar R, eds. *Human Systems Engineering and Design. IHSED 2018. Advances in Intelligent Systems and Computing, Vol 876.* Cham: Springer International Publishing; 2019:287-292.
189. Pazart L, Sall FS, De Luca A, et al. Consideration of the Human Factor in the Design and Development of a New Medical Device: Example of a Device to Assist Manual Ventilation.

Proc 10th Int Jt Conf Biomed Eng Syst Technol. 2017:215-223. doi:10.5220/0006250102150223.

190. Pearson J, Walsh N, Carter D, Koskela S, Hurley M. Developing a Web-Based Version of An Exercise-Based Rehabilitation Program for People With Chronic Knee and Hip Pain: A Mixed Methods Study. *JMIR Res Protoc*. 2016;5(2):1-15. doi:10.2196/resprot.5446.

191. Piau A, Charlon Y, Campo E, Vellas B, Nourhashemi F. A smart insole to promote healthy aging for frail elderly individuals: Specifications, design, and preliminary results. *JMIR Rehabil Assist Technol*. 2015;2(1):1-11. doi:10.2196/rehab.4084.

192. Pilco H, Sanchez-Gordon S, Calle-Jimenez T, et al. An Agile Approach to Improve the Usability of a Physical Telerehabilitation Platform. *Appl Sci*. 2019;9(3). doi:10.3390/app9030480.

193. Possemato K, Kuhn E, Johnson EM, Hoffman JE, Brooks E. Development and refinement of a clinician intervention to facilitate primary care patient use of the PTSD Coach app. *Transl Behav Med*. 2017;7(1):116-126. doi:10.1007/s13142-016-0393-9.

194. Pramana G, Parmanto B, Kendall PC, Silk JS. The SmartCAT: an m-health platform for ecological momentary intervention in child anxiety treatment. *Telemed J e-health Off J Am Telemed Assoc*. 2014;20(5):419-427. doi:10.1089/tmj.2013.0214.

195. Prapkree L, Sadjadi M, Huffman F, Palacios C. Development and pilot testing of the snackability smartphone application to identify healthy and unhealthy snacks. *Healthc Inform Res*. 2019;25(3):161-172. doi:10.4258/hir.2019.25.3.161.

196. Prince RM, Soung Yee A, Parente L, et al. User-Centered Design of a Web-Based Tool to Support Management of Chemotherapy-Related Toxicities in Cancer Patients. *J Med Internet Res*. 2019;21(3):e9958. doi:10.2196/jmir.9958.

197. Prokhorov A V., Machado TC, Calabro KS, et al. Developing mobile phone text messages for tobacco risk communication among college students: a mixed methods study. *BMC Public Health*. 2017;17(1):1-8. doi:10.1186/s12889-017-4027-z.

198. Puijk-Hekman S, van Gaal BG, Bredie SJ, Nijhuis-van der Sanden MW, van Dulmen S. Self-Management Support Program for Patients With Cardiovascular Diseases: User-Centered Development of the Tailored, Web-Based Program Vascular View. *JMIR Res Protoc*. 2017;6(2):1-13. doi:10.2196/resprot.6352.

199. Qin YH, Zhou RY, Wu Q, et al. The effect or nursing participation in the design of a critical care information system: a case study in a Chinese hospital. *BMC Med Inform Decis Mak*. 2017;17(1):165. doi:10.1186/s12911-017-0569-3.

200. Rasmussen G, Bech LL, Nielsen TW. An Applicator Delivery System for Fixed-Combination Calcipotriene Plus Betamethasone Dipropionate Topical Suspension (Gel): Innovating Psoriasis Vulgaris Treatment Through Patient Collaboration. *Dermatol Ther (Heidelb)*. 2015;5(4):235-246. doi:10.1007/s13555-015-0087-0.

201. Realpe A, Elahi F, Bucci S, et al. Co-designing a virtual world with young people to deliver social cognition therapy in early psychosis. *Early Interv Psychiatry*. 2020;14(1):37-43. doi:10.1111/eip.12804.

202. Redwood T, Neill S, Church S, Spencer M. Early Help in Early Years: Developing a Universal Assessment Tool. *Compr Child Adolesc Nurs*. 2019;42(3):190-202. doi:10.1080/24694193.2018.1457104.

203. Reese JM, Joseph RP, Cherrington A, Allison J. Development of Participant-Informed Text Messages to Promote Physical Activity Among African American Women Attending College: A Qualitative Mixed-Methods Inquiry. *J Transcult Nursering*. 2018;28(3):236-242. doi:10.1177/1043659616644959.Development.

204. Revenäs Å, Opava CH, Martin C, Demmelmaier I, Keller C, Åsenlöf P. Development of a Web-Based and Mobile App to Support Physical Activity in Individuals With Rheumatoid Arthritis: Results From the Second Step of a Co-Design Process. *JMIR Res Protoc*. 2015;4(1):1-14. doi:10.2196/resprot.3795.

205. Ribigan A, Badea R, Rusu O, Bajenaru O, Antochi F. FOLLOW.ME – a system dedicated to patients with cognitive impairment. *Rom J Neurol Rev Rom Neurol*. 2018;17:139-143. doi:10.37897/RJN.2018.3.3.

206. Rivera J, McPherson AC, Hamilton J, et al. User-Centered Design of a Mobile App for Weight and Health Management in Adolescents with Complex Health Needs: Qualitative Study. *JMIR Form Res*. 2018;2(1):1-15. doi:10.2196/formative.8248.

207. Rogers J, Spina N, Neese A, Hess R, Brodke D, Lex A. Composer: Visual cohort analysis of patient outcomes. *Appl Clin Inform*. 2018;10(2):278-285.

208. Rohde A, Duensing A, Dawczynski C, Godemann J, Lorkowski S, Brombach C. An app to improve eating habits of adolescents and young adults (Challenge to go): Systematic development of a theory-based and target group–adapted mobile app intervention. *JMIR mHealth uHealth*. 2019;7(8):1-18. doi:10.2196/11575.

209. Rothgangel A, Braun S, Smeets R, Beurskens A. Design and Development of a Telerehabilitation Platform for Patients With Phantom Limb Pain: A User-Centered Approach. *JMIR Rehabil Assist Technol*. 2017;4(1):1-15. doi:10.2196/rehab.6761.

210. Runaas L, Hanauer D, Maher M, et al. BMT Roadmap: A User-Centered Design Health Information Technology Tool to Promote Patient-Centered Care in Pediatric Hematopoietic Cell Transplantation. *Biol blood marrow Transplant J Am Soc Blood Marrow Transplant*. 2017;23(5):813-819. doi:10.1016/j.bbmt.2017.01.080.

211. Salgado TM, Fedrigon A, Omichinski DR, Meade MA, Farris KB. Identifying Medication Management Smartphone App Features Suitable for Young Adults With Developmental Disabilities: Delphi Consensus Study. *JMIR mHealth uHealth*. 2018;6(5). doi:10.2196/mhealth.9527.

212. Sandhu H, Wilson K, Reed N, et al. A Mobile Phone App for the Self-Management of Pediatric Concussion: Development and Usability Testing. *JMIR Hum Factors*. 2019;6(2):e12135. doi:10.2196/12135.

213. Sauer M, Abbotts C. A new pen device for injection of recombinant human growth hormone: a convenience, functionality and usability evaluation study. *Patient Prefer Adherence*. 2018;12:27-34. doi:10.2147/PPA.S149412.

214. Schnall R, Cho H, Mangone A, Pichon A, Jia H. Mobile Health Technology for Improving Symptom Management in Low Income Persons Living with HIV. *AIDS Behav*. 2018;22(10):3373-3383. doi:10.1007/s10461-017-2014-0.

215. Schneider T, Panzera AD, Couluris M, Lindenberger J, McDermott R, Bryant CA. Engaging Teens with Asthma in Designing a Patient-Centered Mobile App to Aid Disease Self-Management. *Telemed e-Health*. 2016;22(2):170-175. doi:10.1089/tmj.2015.0041.

216. Schwartz LA, Psihogios AM, Henry-Moss D, et al. Iterative Development of a Tailored mHealth Intervention for Adolescent and Young Adult Survivors of Childhood Cancer. *Clin Pract Pediatr Psychol*. 2019;7(1):31-43. doi:10.1037/cpp0000272.

217. Sedlmayr B, Schöffler J, Prokosch HU, et al. User-centered design of a mobile medication management. *Informatics Heal Soc Care*. 2019;44(2):152-163. doi:10.1080/17538157.2018.1437042.

218. Sewitch MJ, Fallone CA, Ghali P, Lee GE. What patients want in a smartphone app that supports colonoscopy preparation: Qualitative study to inform a user-centered smartphone app. *JMIR mHealth uHealth*. 2019;7(7):1-10. doi:10.2196/12242.

219. Shellmer DA, Dew MA, Mazariegos G, DeVito Dabbs A. Development and field testing of Teen Pocket PATH(®), a mobile health application to improve medication adherence in adolescent solid organ recipients. *Pediatr Transplant*. 2016;20(1):130-140. doi:10.1111/petr.12639.

220. Shepherd M, Fleming T, Lucassen M, Stasiak K, Lambie I, Merry SN. The design and relevance of a computerized gamified depression therapy program for indigenous m ori adolescents. *JMIR Serious Games*. 2015;3(1):1-13. doi:10.2196/games.3804.

221. Sivan M, Gallagher J, Holt R, Weightman A, O'Connor R, Levesley M. Employing the International Classification of Functioning, Disability and Health framework to capture user feedback in the design and testing stage of development of home-based arm rehabilitation technology. *Assist Technol*. 2016;28(3):175-182. doi:10.1080/10400435.2016.1140689.

222. Smaradottir B, Gerdes M, Martinez S, Fensli R. The EU-project United4Health: User-centred design of an information system for a Norwegian telemedicine service. *J Telemed Telecare*. 2016;22(7):422-429. doi:10.1177/1357633X15615048.

223. Smith-Turchyn J, Gravesande J, Agarwal G, et al. A healthy lifestyle app for older adults with diabetes and hypertension: usability assessment. *Int J Healthc Technol Manag*. 2017;16(3-4):250-270. doi:10.1504/IJHTM.2017.088862.
224. Sobrinho A, Da Silva LD, Perkusich A, Pinheiro ME, Cunha P. Design and evaluation of a mobile application to assist the self-monitoring of the chronic kidney disease in developing countries. *BMC Med Inform Decis Mak*. 2018;18(7):1-14. doi:10.1186/s12911-018-0587-9.
225. Soomro N, Chhaya M, Soomro M, et al. Design, Development, and Evaluation of an Injury Surveillance App for Cricket: Protocol and Qualitative Study. *JMIR mHealth uHealth*. 2019;7(1). doi:10.2196/10978.
226. Srinivas P, Cornet V, Holden R. Human factors analysis, design, and evaluation of Engage, a consumer health IT application for geriatric heart failure self- care. *Int J Hum Comput Interact*. 2017;33(4):298-312. doi:10.1080/10447318.2016.1265784.Human.
227. Stålberg A, Sandberg A, Söderbäck M, Larsson T. The child’s perspective as a guiding principle: Young children as co-designers in the design of an interactive application meant to facilitate participation in healthcare situations. *J Biomed Inform*. 2016;61:149-158. doi:10.1016/j.jbi.2016.03.024.
228. Steele Gray C, Khan AI, Kuluski K, et al. Improving Patient Experience and Primary Care Quality for Patients With Complex Chronic Disease Using the Electronic Patient-Reported Outcomes Tool: Adopting Qualitative Methods Into a User-Centered Design Approach. *JMIR Res Protoc*. 2016;5(1):e28. doi:10.2196/resprot.5204.
229. Stinson JN, Lallo C, Harris L, et al. iCanCope with Pain™: User-centred design of a web- and mobile-based self-management program for youth with chronic pain based on identified health care needs. *Pain Res Manag*. 2014;19(5):257-266.
230. Tamblyn R, Winslade N, Lee TC, et al. Improving patient safety and efficiency of medication reconciliation through the development and adoption of a computer-assisted tool with automated electronic integration of population-based community drug data: The RightRx project. *J Am Med Informatics Assoc*. 2018;25(5):482-495. doi:10.1093/JAMIA/OCX107.
231. Teh RCA, Visvanathan R, Ranasinghe D, Wilson A. Evaluation and refinement of a handheld health information technology tool to support the timely update of bedside visual cues to prevent falls in hospitals. *Int J Evid Based Healthc*. 2018;16(2):90-100. doi:10.1097/XEB.0000000000000129.
232. Terp M, Jørgensen R, Laursen BS, Mainz J, Bjørnes CD. A smartphone app to foster power in the everyday management of living with schizophrenia: Qualitative analysis of young adults’ perspectives. *J Med Internet Res*. 2018;5(4):1-14. doi:10.2196/10157.
233. Thilo FJSS, Hahn S, Halfens RJGG, Schols JJMGA. Usability of a wearable fall detection prototype from the perspective of older people-A real field testing approach. *J Clin Nur*. 2019;28(1):310-320. doi:10.1111/jocn.14599.

234. Thirumalai M, Rimmer JH, Johnson G, et al. Teams (Tele-exercise and multiple sclerosis), a tailored telerehabilitation mhealth app: Participant-centered development and usability study. *JMIR mHealth uHealth*. 2018;6(5):1-14. doi:10.2196/10181.
235. Thomas S, Pulman A, Thomas P, et al. Digitizing a Face-to-Face Group Fatigue Management Program: Exploring the Views of People With Multiple Sclerosis and Health Care Professionals Via Consultation Groups and Interviews. *JMIR Form Res*. 22AD;3(2):e10951. doi:10.2196/10951.
236. Threatt AL, Merino J, Brooks JO, et al. The design, prototyping, and formative evaluation of an assistive robotic table (ART) for stroke patients. *Heal Environ Res Des J*. 2017;10(3):152-169. doi:10.1177/1937586716687802.
237. Timmerman JG, Tönis TM, Dekker-Van Weering MGH, et al. Co-creation of an ICT-supported cancer rehabilitation application for resected lung cancer survivors: Design and evaluation. *BMC Health Serv Res*. 2016;16(155):1-11. doi:10.1186/s12913-016-1385-7.
238. Timmons SE, Shakibnia EB, Gold MA, Garbers S. MyLARC: A Theory-Based Interactive Smartphone App to Support Adolescents' Use of Long-Acting Reversible Contraception. *J Pediatr Adolesc Gynecol*. 2018;31(3):285-290. doi:10.1016/j.jpag.2017.11.005.
239. Toefy Y, Skinner D, Thomsen S. "Please Don't Send Us Spam!" A Participative, Theory-Based Methodology for Developing an mHealth Intervention. *JMIR mHealth uHealth*. 2016;4(3). doi:10.2196/mhealth.6041.
240. Tongpeth J, Du HY, Clark RA. Development and feasibility testing of an avatar-based education application for patients with acute coronary syndrome. *J Clin Nurs*. 2018;27(19-20):3561-3571. doi:10.1111/jocn.14528.
241. Tonheim AN, Babic A. Multiple Sclerosis Application Design with Medical Expert Evaluation. *World Congr Med Phys Biomed Eng 2018*. 2019;68:457-461. doi:10.1007/978-981-10-9035-6_84.
242. Tonkin E, Jeffs L, Wycherley TP, et al. A smartphone app to reduce sugar-sweetened beverage consumption among young adults in Australian remote indigenous communities: Design, formative evaluation and user-testing. *JMIR mHealth uHealth*. 2017;5(12):1-17. doi:10.2196/mhealth.8651.
243. Torres IG, Parmar G, Aggarwal S, Mansur N, Guthrie A, Machinery AC. Affordable Smart Wheelchair. *Ext Abstr 2019 CHI Conf*. 2019;(CHI Conference on Human Factors in Computing Systems (CHI)). doi:10.1145/3290607.3308463.
244. Tucker Edmonds B, Hoffman SM, Lynch D, et al. Creation of a Decision Support Tool for Expectant Parents Facing Threatened Periviable Delivery: Application of a User-Centered Design Approach. *Patient*. 2019;12(3):327-337. doi:10.1007/s40271-018-0348-y.

245. Vácha T, Kandusová V. Making innovation in elderly care possible using participatory design: the smart home-care project in Prague. In: *2018 Smart City Symposium Prague (SCSP)*. ; 2018:1-6. doi:10.1109/SCSP.2018.8402671.

246. van der Velden M, Sommervold MM, Culén A, Nakstad B. Designing Interactive Technologies with Teenagers in a Hospital Setting BT - Perspectives on HCI Research with Teenagers. In: Little L, Fitton D, Bell BT, Toth N, eds. *Perspectives on HCI Research with Teenagers. Human–Computer Interaction Series.* Cham: Springer International Publishing; 2016:103-131. doi:10.1007/978-3-319-33450-9_5.

247. Vanoh D, Ishak IH, Shahar S, Manaf ZA, Ali NM, Noah SAM. Development and assessment of a web-based intervention for educating older people on strategies promoting healthy cognition. *Clin Interv Aging*. 2018;13:1787-1798. doi:10.2147/CIA.S157324.

248. Verbiest MEA, Corrigan C, Dalhousie S, et al. Using codesign to develop a culturally tailored, behavior change mHealth intervention for indigenous and other priority communities: A case study in New Zealand. *Transl Behav Med*. 2019;9(4):720-736. doi:10.1093/tbm/iby093.

249. Villegas N, Santisteban D, Cianelli R, et al. The development, feasibility and acceptability of an Internet-based STI-HIV prevention intervention for young Chilean women. *Int Nurs Rev*. 2014;61(1):55-63. doi:10.1111/inr.12080.

250. Voorend R, Derboven J, Slegers K, Assoc Comp M. Distributed User-Generated Card Based Co-Design: A Case-Study. *Ext Abstr 2019 CHI Conf Hum Factors Comput Syst*. 2019:1-6. doi:10.1145/3290607.3312815.

251. Vorrink SNW, Kort HSM, Troosters T, Lammers JWJ. A mobile phone app to stimulate daily physical activity in patients with chronic obstructive pulmonary disease: Development, feasibility and pilot studies. *JMIR mHealth uHealth*. 2016;4(1):1-12. doi:10.2196/mhealth.4741.

252. Wachtler C, Coe A, Davidson S, et al. Development of a mobile clinical prediction tool to estimate future depression severity and guide treatment in primary care: User-centered design. *JMIR mHealth uHealth*. 2018;6(4):1-15. doi:10.2196/mhealth.9502.

253. Walker JG, Bickerstaffe A, Hewabandu N, et al. The CRISP colorectal cancer risk prediction tool: an exploratory study using simulated consultations in Australian primary care. *BMC Med Inform Decis Mak*. 2017;17. doi:10.1186/s12911-017-0407-7.

254. Wang J, Yao N, Wang Y, et al. Developing “Care Assistant”: A smartphone application to support caregivers of children with acute lymphoblastic leukaemia. *J Telemed Telecare*. 2016;22(3):163-171. doi:10.1177/1357633X15594753.

255. Ward G, Fielden S, Muir H, Holliday N, Urwin G. Developing the assistive technology consumer market for people aged 50-70. *Ageing Soc*. 2017;37(5):1050-1067. doi:10.1017/S0144686X16000106.

256. Ward G, Holliday N, Awang D, Harson D. Creative approaches to service design: Using co-creation to develop a consumer focused assistive technology service. *Technol Disabil*. 2015;27:5-15. doi:10.3233/TAD-150424.

257. Ward R, Taha KM. Patient Involvement as Experts in the Development and Assessment of a Smartphone App as a Patient Education Tool for the Management of Thalassemia and Iron Overload Syndromes. *Hemoglobin*. 2016;40(5):323-329. doi:10.1080/03630269.2016.1217875.

258. Warren I, Meads A, Whittaker R, Dobson R, Ameratunga S. Behavior change for youth drivers: design and development of a smartphone-based app (BackPocketDriver). *JMIR Form Res*. 2018;2(2):1-18. doi:10.2196/formative.9660.

259. Waterman L, Szatala S, Motta BD, Son CH, To J, Acm. Mellow: A Mobile Application to Help Youth in Crisis. *Ext Abstr 2018 CHI Conf Hum Factors Comput Syst*. 2018:1-6. doi:10.1145/3170427.3180652.

260. Webster R, Michie S, Estcourt C, Gerressu M, Bailey J V. Increasing condom use in heterosexual men: development of a theory-based interactive digital intervention. *Transl Behav Med*. 2016;6(3):418-427. doi:10.1007/s13142-015-0338-8.

261. Weichelt B, Bendixsen C, Keifer M. Farm Owners and Workers as Key Informants in User-Centered Occupational Health Prototype Development: A Stakeholder-Engaged Project. *J Med Internet Res*. 2019;21(1):e9711. doi:10.2196/jmir.9711.

262. Wentzel J, van Velsen L, van Limburg M, et al. Participatory eHealth development to support nurses in antimicrobial stewardship. *BMC Med Inform Decis Mak*. 2014;14(1):45. doi:10.1186/1472-6947-14-45.

263. Werner-Seidler A, O'Dea B, Shand F, et al. A Smartphone App for Adolescents With Sleep Disturbance: Development of the Sleep Ninja. *JMIR Ment Heal*. 2017;4(3). doi:10.2196/mental.7614.

264. Whiteley L, Mena L, Craker LK, Healy MG, Brown LK. Creating a Theoretically Grounded Gaming App to Increase Adherence to Pre-Exposure Prophylaxis: Lessons From the Development of the Viral Combat Mobile Phone Game. *JMIR Serious Games*. 2019;7(1):e11861. doi:10.2196/11861.

265. Wilcox L, Woollen J, Prey J, et al. Interactive tools for inpatient medication tracking: a multi-phase study with cardiothoracic surgery patients. *J Am Med Inform Assoc*. 2016;23(1):144-158. doi:10.1093/jamia/ocv160.

266. Willard S, Cremers G, Man YP, Van Rossum E, Spreeuwenberg M, De Witte L. Development and testing of an online community care platform for frail older adults in the Netherlands: A user-centred design. *BMC Geriatr*. 2018;18(87):1-9. doi:10.1186/s12877-018-0774-7.

267. Williamson SS, Gorman PN, Jimison HB. A mobile/web app for long distance caregivers of older adults: functional requirements and design implications from a user centered design process. *AMIA . Annu Symp proceedings AMIA Symp*. 2014;2014:1960-1969.

268. Wilson SM, Thompson AC, Currence ED, et al. Patient-Informed Treatment Development of Behavioral Smoking Cessation for People With Schizophrenia. *Behav Ther*. 2019;50(2):395-409. doi:10.1016/j.beth.2018.07.004.

269. Winterling J, Wiklander M, Obol CM, et al. Development of a Self-Help Web-Based Intervention Targeting Young Cancer Patients With Sexual Problems and Fertility Distress in Collaboration With Patient Research Partners. *JMIR Res Protoc*. 2016;5(2):1-12. doi:10.2196/resprot.5499.

270. Wolpin SE, Halpenny B, Whitman G, et al. Development and usability testing of a web-based cancer symptom and quality-of-life support intervention. *Health Informatics J*. 2015;21(1):10-23. doi:10.1177/1460458213495744.

271. Woods L, Duff J, Roehrer E, Walker K, Cummings E. Design of a Consumer Mobile Health App for Heart Failure: Findings From the Nurse-Led Co-Design of Care4myHeart. *JMIR Nurs*. 2019;2(1):e14633. doi:10.2196/14633.

272. Wozney L, Baxter P, Newton AS. Usability evaluation with mental health professionals and young people to develop an Internet-based cognitive-behaviour therapy program for adolescents with anxiety disorders. *BMC Pediatr*. 2015;15. doi:10.1186/s12887-015-0534-1.

273. Yoo S, Lee K-H, Baek H, et al. Development and User Research of a Smart Bedside Station System toward Patient-Centered Healthcare System. *J Med Syst*. 2015;39(9):86. doi:10.1007/s10916-015-0273-8.

274. Zachary W, Michlig G, Kaplan A, Nguyen N-T, Quinn CC, Surkan PJ. Participatory Design of a Social Networking App to Support Type II Diabetes Self-Management in Low-Income Minority Communities. *Proc Int Symp Hum Factors Erg Heal*. 2017;6(1):37-43. doi:10.1177/2327857917061010.Participatory.

275. Zafeiridi P, Paulson K, Dunn R, et al. A Web-Based Platform for People with Memory Problems and Their Caregivers (CAREGIVERSPRO-MMD):Mixed-Methods Evaluation of Usability. *JMIR Form Res*. 2018;2(1):1-12. doi:10.2196/formative.9083.

276. Zieve GG, Richardson LP, Katzman K, Spielvogle H, Whitehouse S, McCarty CA. Adolescents’ perspectives on personalized e-feedback in the context of health risk behavior screening for primary care: Qualitative study. *J Med Internet Res*. 2017;19(7):1-11. doi:10.2196/jmir.7474.

277. Zuidema RM, van Gaal BG, van Dulmen S, Repping-Wuts H, Schoonhoven L. An Online Tailored Self-Management Program for Patients With Rheumatoid Arthritis: A Developmental Study. *JMIR Res Protoc*. 2015;4(4):1-9. doi:10.2196/resprot.4571.
